# Potentiating the radiation-induced type I interferon antitumoral immune response by ATM inhibition in pancreatic cancer

**DOI:** 10.1172/jci.insight.168824

**Published:** 2024-02-20

**Authors:** Qiang Zhang, Long Jiang, Weiwei Wang, Amanda K. Huber, Victoria M. Valvo, Kassidy M. Jungles, Erin A. Holcomb, Ashley N. Pearson, Stephanie The, Zhuwen Wang, Leslie A. Parsels, Joshua D. Parsels, Daniel R. Wahl, Arvind Rao, Vaibhav Sahai, Theodore S. Lawrence, Michael D. Green, Meredith A. Morgan

**Affiliations:** 1 Department of Radiation Oncology and; 2 Rogel Cancer Center, University of Michigan, Ann Arbor, Michigan, USA.; 3 Department of Pathology, The First Affiliated Hospital of Zhengzhou University, Zhengzhou, China.; 4 Department of Pharmacology, University of Michigan, Ann Arbor, Michigan, USA.; 5 Department of Biostatistics, University of Michigan School of Public Health, Ann Arbor, Michigan, USA.; 6 Division of Hematology and Oncology, Department of Internal Medicine, and; 7 Department of Microbiology and Immunology, University of Michigan, Ann Arbor, Michigan, USA.; 8 Department of Radiation Oncology, Veterans Affairs Ann Arbor Healthcare System, Ann Arbor, Michigan, USA.

**Keywords:** Oncology, DNA repair, Innate immunity, Radiation therapy

## Abstract

Radiotherapy induces a type I interferon–mediated (T1IFN-mediated) antitumoral immune response that we hypothesized could be potentiated by a first-in-class ataxia telangiectasia mutated (ATM) inhibitor, leading to enhanced innate immune signaling, T1IFN expression, and sensitization to immunotherapy in pancreatic cancer. We evaluated the effects of AZD1390 or a structurally related compound, AZD0156, on innate immune signaling and found that both inhibitors enhanced radiation-induced T1IFN expression via the POLIII/RIG-I/MAVS pathway. In immunocompetent syngeneic mouse models of pancreatic cancer, ATM inhibitor enhanced radiation-induced antitumoral immune responses and sensitized tumors to anti–PD-L1, producing immunogenic memory and durable tumor control. Therapeutic responses were associated with increased intratumoral CD8^+^ T cell frequency and effector function. Tumor control was dependent on CD8^+^ T cells, as therapeutic efficacy was blunted in CD8^+^ T cell–depleted mice. Adaptive immune responses to combination therapy provided systemic control of contralateral tumors outside of the radiation field. Taken together, we show that a clinical candidate ATM inhibitor enhances radiation-induced T1IFN, leading to both innate and subsequent adaptive antitumoral immune responses and sensitization of otherwise resistant pancreatic cancer to immunotherapy.

## Abstract 2

A recently developed ATM inhibitor enhances radiation-induced T1IFN, leading to both innate and subsequent adaptive anti-tumoral immune responses and sensitization of immunotherapy-resistant pancreatic cancer.

## Graphical abstract

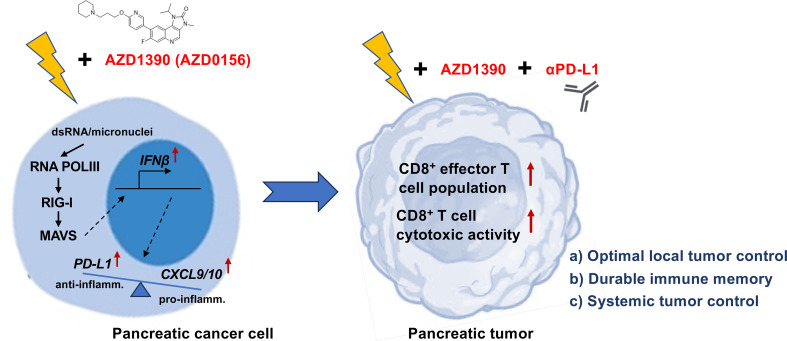

## Introduction

Pancreatic cancer is refractory to immunotherapy (1). Standard of care for locally advanced pancreatic cancer is multiagent chemotherapy and chemoradiation. Despite improvements in systemic therapy over the last decade and the ability of radiation to improve local disease, survival is still poor (2, 3). Given the importance of local disease management to overall survival and the presence of occult metastatic disease in the majority of these patients, further improvements in overall survival require efficacious therapies against both local and systemic disease (4).

Radiation can elicit a type I interferon (T1IFN) response that involves the release of damaged DNA from the nucleus as micronuclei or cytosolic double-strand DNA (dsDNA). Sensing of cytoplasmic DNA is mediated by cyclic GMP-AMP (cGAS) and stimulator of interferon gene (STING) (5, 6). Cytosolic DNA may be converted to RNA by RNA polymerase III (POLIII), generating cytosolic RNA that, together with mitochondrial RNA, is sensed by the retinoic acid–inducible gene I (RIG-I)/mitochondrial antiviral-signaling protein (MAVS) pathway (7, 8). The cGAS/STING and RIG-I/MAVS pathways both activate downstream TANK-binding kinase 1 (TBK1) and the transcription factor IRF3, ultimately leading to T1IFN production (9, 10). Recent studies demonstrated that the cancer cell type and species (human versus mouse) contribute to the relative contributions of the cGAS/STING and POLIII/RIG-I/MAVS nucleic acid–sensing pathways in radiation-induced T1IFN expression (8, 11). Although our prior study (12) suggested that radiation and the DNA-dependent protein kinase (DNA-PK) inhibitor peposertib (M3814) promote T1IFN induction via a POLIII/RIG-I/MAVS-dependent manner, it is unknown whether this is a common pathway connecting the radiation-induced cytosolic nucleic acids and T1IFN in pancreatic cancer cells.

The T1IFN response to radiation contributes to the synergy between radiation and immunotherapy in some cancers (13). While pancreatic cancer is resistant to treatment with immunotherapy alone, limited clinical data do suggest that combining radiation with immunotherapy is safe and may have modest activity in patients with metastatic pancreatic ductal adenocarcinoma (14, 15). To further improve upon the ability of radiation to induce T1IFN responses and synergize with immunotherapy, additional therapeutic combinations are warranted. As such, inhibitors of the DNA damage response are an attractive strategy, given their ability to cause persistence of radiation-induced DNA damage and, in theory, immunogenic DNA damage, leading to a T1IFN-mediated antitumoral immune response. For example, recent studies from our group and others have shown that inhibitors of DNA-PK and ataxia telangiectasia and rad3 related (ATR) enhance radiation-induced T1IFN antitumor immune responses (8, 12, 16).

Another promising target for enhancing both radiosensitization and the radiation-induced innate immune response is ataxia telangiectasia mutated (ATM), a master regulator of the DNA damage response to radiation-induced double-strand breaks (17, 18). ATM negatively regulates the viral-mediated innate immune response as well as cytoplasmic leakage of mitochondrial DNA (9, 19). Our prior study revealed a role for ATM in restraining the radiation-induced innate immune response in tumors (20). While this study demonstrated that genetic silencing of ATM enhanced radiation-induced T1IFN and promoted antitumoral immune responses, it remains unknown if pharmacologic inhibitors of ATM will have the same effect. AZD1390 is a first-in-class ATM inhibitor that radiosensitizes in preclinical tumor models (21) and is under investigation in combination with radiation in phase I clinical trials (NCT03423628, NCT05116254). The effects of AZD1390 on radiation-induced T1IFN or the antitumor immune response are currently unknown.

Therefore, in this study we sought to test the hypothesis that ATM inhibition by AZD1390 (or a structurally related compound, AZD0156) enhances radiation-induced T1IFN responses and sensitizes otherwise resistant pancreatic cancer to immunotherapy, inducing both innate and CD8^+^ T cell–dependent adaptive immune responses. To test this hypothesis, we assessed T1IFN responses following treatment with AZD1390 or AZD0156 and radiation, including mechanistic studies to ascertain the contribution of the cGAS/STING and POLIII/RIG-I/MAVS pathways as well as interferon-stimulated gene expression. Furthermore, we investigated therapeutic efficacy in vivo in immune-competent mouse models of pancreatic cancer and the ability of AZD1390 and radiation to sensitize both primary and unirradiated contralateral tumors to PD-L1 immunotherapy. In addition, we characterized the effects of therapy on the tumor immune microenvironment. Overall, this study forms the foundation for future clinical investigations combining ATM inhibitors with radiation and immunotherapy with the goal of improving both local and systemic disease management in patients with pancreatic cancer.

## Results

### ATM inhibitor enhances radiation-induced T1IFN expression and signaling.

**Figure 1:**
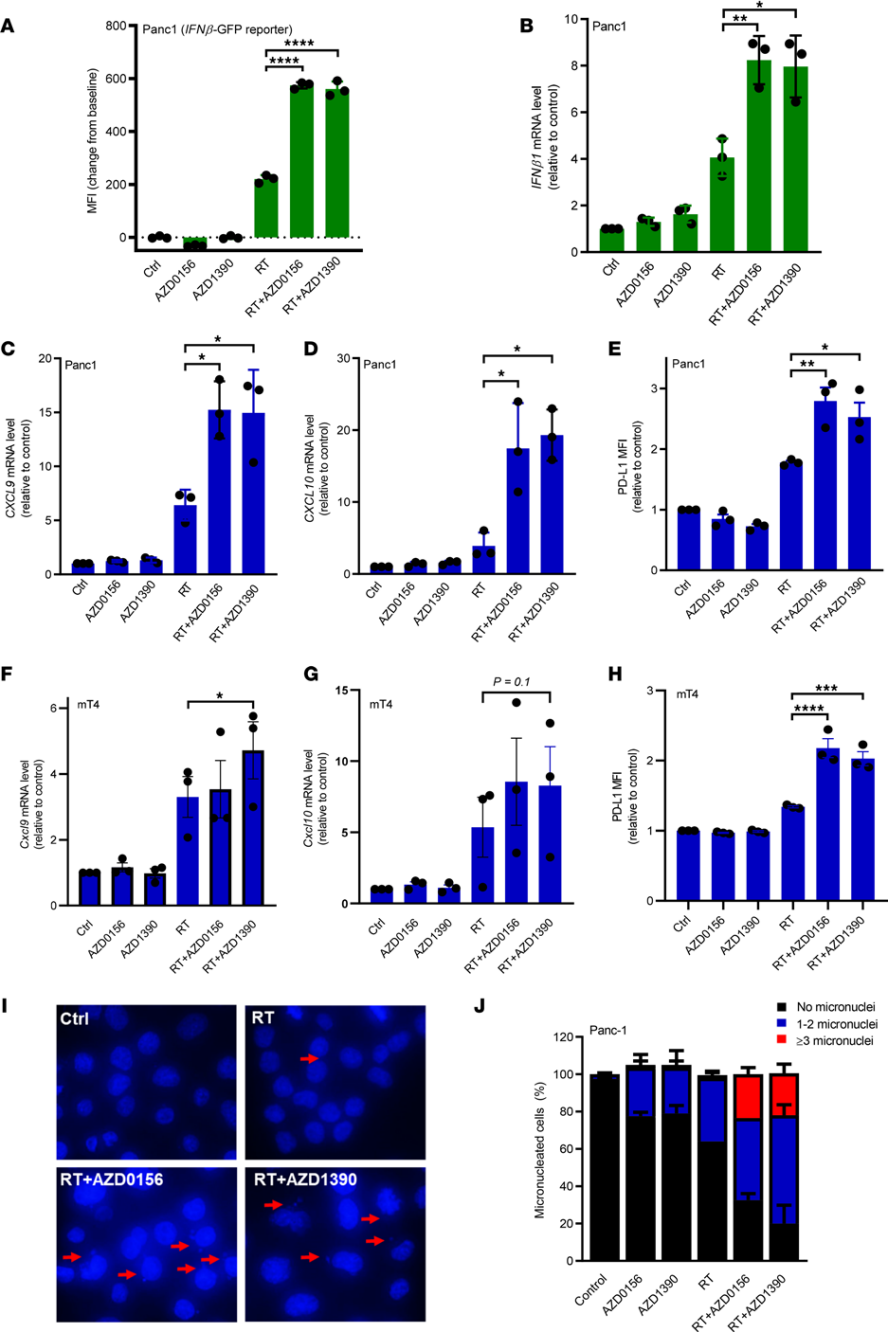
AZD1390 or AZD0156 promotes radiation-induced T1IFN expression and signaling. Pancreatic cancer cells were treated with AZD0156 (30 nM) or AZD1390 (30 nM) 1 hour before radiation (RT; 8 Gy) and analyzed at 3 days after radiation. (**A**) Panc1 cells with stable *IFNβ1* promoter–GFP reporter were treated as indicated and assessed for MFI of GFP expression. (**B**) qPCR for *IFNβ1* mRNA in treated Panc1 cells. (**C** and **D**) qPCR analysis of mRNA levels of interferon-stimulated genes *CXCL9* (**C**) and *CXCL10* (**D**) in treated Panc1 cells. (**E**) Flow cytometry analysis of cell surface PD-L1 expression in Panc1 cells following the indicated treatments. Data represent the MFI for PD-L1 minus the MFI for isotype control and are shown as relative fold change. (**F** and **G**) qPCR analysis of mRNA levels of interferon-stimulated genes *Cxcl9* (**F**) and *Cxcl10* (**G**) in mouse mT4 cells. (**H**) Flow cytometry analysis of cell surface PD-L1 expression in mT4 cells treated as indicated. Data represent the MFI for PD-L1 minus the MFI for isotype control and are shown as relative fold change. (**I**) Representative DAPI immunofluorescence of Panc1 cells, with arrows indicating micronuclei. (**J**) Percentage of Panc1 cells that contain different numbers of micronuclei (0, 1–2, or ≥3) in each treatment condition in **I**. Error bars represent the SD of 2 independent experiments. (**A**–**H**) Data are expressed as the mean ± SEM (*n* = 3 independent experiments with each performed in technical triplicate). Statistical analyses were carried out by 1-way ANOVA with a multiple comparison post test. *P < 0.05, **P < 0.01, ***P < 0.001, ****P < 0.0001.

Induction of T1IFN is essential for the antitumor immune effects of radiation (22). We hypothesized that pharmacologic inhibition of ATM, the apical kinase in DNA damage response, would further increase T1IFN and antitumor immune responses. To test this hypothesis, Panc1 cells stably expressing a GFP reporter driven by the human *IFNβ1* promoter (5, 20) were treated with AZD1390, AZD0156, and/or radiation. Radiation, but neither ATM inhibitor, modestly induced *IFNβ1* promoter–driven GFP expression. The combination of either ATM inhibitor and radiotherapy further increased T1IFN reporter activity (Figure 1A). To confirm this finding, *T1IFN* mRNA levels were measured in Panc1 cells following treatment. We observed a significant increase in endogenous *IFNB1* mRNA in Panc1 cells treated with radiation and ATM inhibitor (Figure 1B). We next investigated whether radiation and ATM inhibitor modulated the expression of the interferon-stimulated genes *CXCL9* and *CXCL10*. Consistent with prior studies (20, 23), radiation increased expression of these interferon-stimulated genes (Figure 1, C and D), and this induction was significantly increased by ATM inhibitor. As PD-L1 is an interferon response gene, we investigated whether the combination of radiation and AZD1390 or AZD0156 would promote PD-L1 expression. We found that treatment with ATM inhibitors alone did not affect PD-L1 expression, while radiation increased cell surface PD-L1 levels (Figure 1E). ATM inhibitor in combination with radiation significantly increased PD-L1 expression compared with radiation alone in Panc1 cells. The ability of combined treatment with AZD1390 and radiation to enhance interferon-stimulated gene *Cxcl9*, *Cxcl10*, and *PD-L1* expression was confirmed in two murine pancreatic adenocarcinoma lines, mT4 and KPC2 (Figure 1, F–H, and Supplemental Figure 1, A–C; supplemental material available online with this article; https://doi.org/10.1172/jci.insight.168824DS1).

Micronuclei are formed in response to radiation-induced DNA damage via the loss of acentric chromosome fragments during mitosis and are a source of cytosolic DNA that activates innate immune signaling leading to T1IFN production (5, 24). To test whether ATM inhibitors could increase micronuclei formation following radiation, Panc1 cells were treated with radiation and AZD0156 or AZD1390. Treatment with radiation or ATM inhibitor alone caused a modest increase in the proportion of micronucleated cells (Figure 1I). The combination of either AZD1390 or AZD0156 with radiation increased both the proportion of Panc1 cells with micronuclei as well as the frequency of micronuclei within cells (Figure 1, I and J).

The cytosolic dsDNA sensor cGAS and its adaptor STING have been implicated in the activation of TBK1 in response to damaged DNA induced by both radiation and DNA damage response inhibition (25–27). To investigate the contribution of cGAS/STING signaling to T1IFN production and signaling in response to radiation and ATM inhibitor, we generated CRISPR/Cas9-mediated cGAS- and STING-KO Panc1 cells. First, we found that cGAS- or STING-KO could block TBK1 phosphorylation by herring testis DNA (HT-DNA), indicating the function of cGAS and STING is intact in Panc1 cells (Supplemental Figure 1D). Surprisingly, neither cGAS nor STING deletion in Panc1 cells significantly affected *IFNβ1* reporter activity in response to radiation alone or the combination with AZD1390 or AZD0156 (Supplemental Figure 1E). Deletion of cGAS and STING also failed to attenuate *IFNβ1* or PD-L1 induction following radiation and AZD1390 or AZD0156 treatment (Supplemental Figure 1, F and G). TBK1 integrates multiple innate immune sensors to induce T1IFN (28). We found that TBK1 was required for the enhancement of *IFNβ1* promoter activity following combined treatment with radiation and ATM inhibitor (Supplemental Figure 1H). TBK1 deletion also eliminated the effects of combined treatment with radiation and ATM inhibitor on *IFNB1* mRNA expression (Supplemental Figure 1I) and PD-L1 cell surface expression (Supplemental Figure 1J). These data suggest that radiation and ATM inhibitor activate TBK1 and T1IFN signaling in a cGAS/STING-independent manner. We further extended our observations to other DNA damage response inhibitors, including the DNA-PK inhibitor M3814 (peposertib) and the ATR inhibitor AZD6738 (ceralasertib), and found that, similar to AZD1390, neither of the inhibitors in conjunction with radiation induced T1IFN production via the cGAS/STING pathway (Supplemental Figure 1K), further supporting that the cGAS/STING cytosolic dsDNA sensing pathway is dispensable for T1IFN signaling induced by ATM inhibitor and radiation in pancreatic cancer.

### ATM inhibitor and radiation activate T1IFN signaling in a POLIII/RIG-I/MAVS–dependent manner.

**Figure 2:**
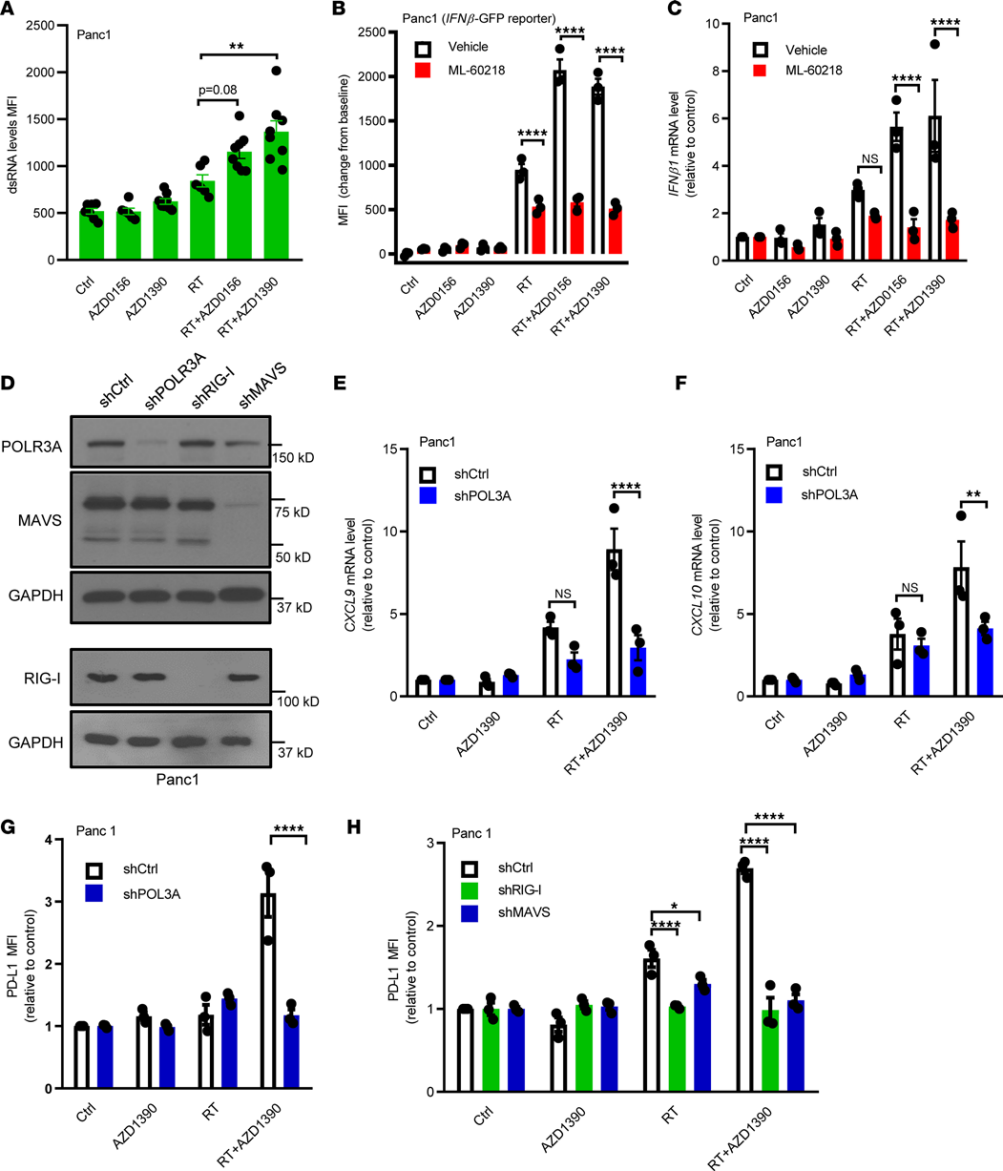
ATM inhibitor and radiation activate T1IFN signaling in a POLIII/RIG-I/MAVS–dependent manner. (**A**) Flow cytometry analysis of cellular dsRNA levels in Panc1 cells at 3 days after the indicated treatments. Data represent the MFI for dsRNA minus the MFI for isotype control and are expressed as the mean ± SEM. (**B**) Panc1 cells stably expressing *IFNβ1*-promoter GFP reporter were treated with radiation (RT; 8 Gy) and/or AZD0156 (30 nM) or AZD1390 (30 nM) in the absence or presence of the POLIII inhibitor ML-60218 (20 μM per day for 3 days). GFP expression levels were determined at day 3 by flow cytometry. (**C**) Panc1 cells were treated as indicated, and *IFNβ1* mRNA levels were measured by qPCR. (**D**) Western blots showing the expression of POLR3A, RIG-I, and MAVS in Panc1 cells with indicated shRNA. (**E** and **F**) qPCR analysis of *CXCL9* (**E**) and *CXCL10* (**F**) mRNA levels in POL3A-depleted Panc1 cells treated as indicated at day 3. (**G**) Relative cell surface PD-L1 expression of shCtrl and shPOL3A Panc1 cells was measured by flow cytometry following the indicated treatments at day 3. (**H**) Cell surface PD-L1 in Panc1 cells (shCtrl, shMAVS, and shRIG-I) at 3 days following the indicated treatments. In **B**, **C**, and **E**–**H**, data are expressed as the mean ± SEM (*n* = 3 independent experiments with each performed in technical triplicate). Statistical analyses were carried out by 1-way ANOVA with a multiple comparison post test. *P < 0.05, **P < 0.01, ****P < 0.0001.

TBK1 functions as a central node for several innate immune pathways, including the POLIII/RIG-I/MAVS pathway, which is required for radiation-induced T1IFN expression in some cancer types (29–31). Given that the POLIII/RIG-I/MAVS pathway is activated by RNA, we first tested the generation of cytosolic double-strand RNA (dsRNA) in Panc1 cells treated with radiation and AZD0156 or AZD1390. We found little effect of radiation alone on dsRNA but a significant increase in dsRNA levels in response to combined treatment with radiation and AZD0156 or AZD1390 (Figure 2A). Additionally, we treated *IFNβ1* promoter reporter Panc1 cells with radiation and/or ATM inhibitor in the presence or absence of ML-60218, a pharmacologic inhibitor of POLIII. Pharmacologic inhibition of POLIII reversed the effects radiation and ATM inhibitor on T1IFN reporter expression (Figure 2B). Consistent with this finding, POLIII inhibition also blocked the induction in *IFNB1* mRNA expression following treatment with radiation and ATM inhibitor (Figure 2C). To confirm this finding, we next silenced POLR3A (the largest subunit of POLIII) in Panc1 cells using shRNA (Figure 2D). Knockdown of POLR3A diminished the effects of radiation and AZD1390 on induction of the interferon response genes *CXCL9* and *CXCL10* (Figure 2, E and F). Inhibition of POLIII also blocked the induction of *Cxcl9* and *Cxcl10* by radiation and AZD1390 in KPC2 cells (Supplemental Figure 2, A and B). To further evaluate the dependence of the interferon-stimulated gene response on POLIII, we examined cell surface PD-L1 expression. Silencing or inhibition of POLR3A prevented induction of PD-L1 by radiation and AZD1390 in Panc1 and murine KPC2 cells (Figure 2G and Supplemental Figure 2, C and D). POLIII-mediated transcription can convert sequence-specific DNA (e.g., AT-rich DNA) to immunogenic RNA and then activate RIG-I/MAVS-dependent interferon signaling (31). RIG-I activation is a multistep process that includes polyubiquitination of multiple lysines within RIG-I (via K63 linkage that is not associated with protein degradation) that are required for a RIG-I activation, downstream signaling, and subsequent T1IFN induction (32, 33). Radiation alone or in combination with AZD0156 or AZD1390 enhanced RIG-I polyubiquitination in Panc1 cells (Supplemental Figure 2E), suggesting a direct involvement of this RNA sensor in the radiation-induced innate immune response. We then examined the effects of radiation and AZD1390 on the innate immune response using PD-L1 as a surrogate in RIG-I– and MAVS-depleted cells (Figure 2D). As expected, silencing of RIG-I or MAVS also diminished PD-L1 cell surface expression following radiation and ATM inhibitor treatment in Panc1 cells (Figure 2H). In addition, knockdown of RIG-I or MAVS had minimal effects on cell cycle progression (Supplemental Figure 2F), supporting a direct involvement of RIG-I/MAVS in T1IFN production that is not indirectly mediated by cell cycle arrest (5, 34). Finally, consistent with our prior study (12), we found that RIG-I/MAVS signaling is required for T1IFN induction in response to DNA-PK inhibitor (M3814) and radiation, similarly to ATM inhibitor and radiation (Supplemental Figure 2G). These data suggest that ATM inhibitor enhances the radiation-induced T1IFN response in a POLIII/RIG-I/MAVS-dependent manner in pancreatic cancer cells.

### Therapeutic targeting of ATM in combination with radiotherapy induces an antitumoral immune response and sensitizes tumors to anti–PD-L1 immunotherapy in pancreatic cancer.

**Figure 3:**
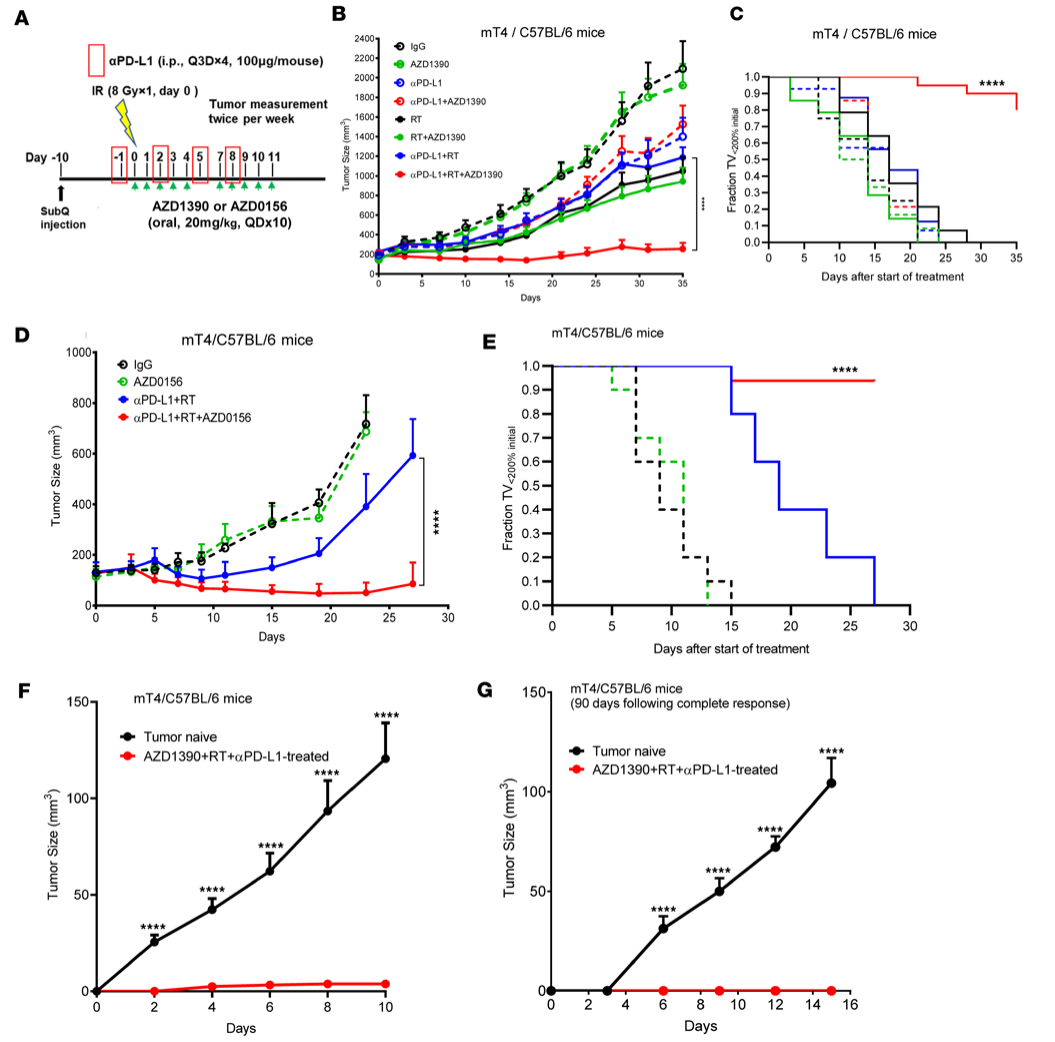
Combined therapy with ATM inhibitor, radiotherapy, and anti–PD-L1 inhibits pancreatic tumor growth and induces durable antitumor immune responses. (**A**) Schematic showing schedules of AZD1390 or AZD0156, radiation (RT), and anti–PD-L1 antibody treatment. AZD1390 or AZD0156 (20 mg/kg) was orally administered approximately 1 hour before radiation (8 Gy) on day 0 as well as on days 1–4 and 7–11. Mouse anti–PD-L1 antibody (100 μg/mL) was intraperitoneally injected every 3 days for a total of 4 doses. (**B** and **C**) C57BL/6 mice with mT4 tumors were treated as illustrated with AZD1390 in **A**. Data represent mean tumor volumes ± SEM (**B**) or tumor volume doubling time (**C**). Data are from *n* = 10 (ctrl), 10 (AZD1390), 12 (αPD-L1), 16 (AZD1390+αPD-L1), 14 (RT), 16 (AZD1390+RT), 16 (αPD-L1+RT), and 20 (AZD1390+RT+αPD-L1) tumors per treatment group. (**D** and **E**) C57BL/6 mice with mT4 tumors were treated with AZD0156 as illustrated in **A**. Data represent mean tumor volumes ± SEM (**D**) or the time for tumor volume doubling (**E**). Data are from *n* = 10–16 tumors per treatment group. (**F** and **G**) Mice with complete responses to AZD1390, RT, and anti–PD-L1 were rechallenged with mT4 (106) cells 7 days (**F**) and 90 days (**G**) after complete response. Naive C57BL/6 were similarly rechallenged. Data represent the mean tumor volume from naive (*n* = 10) or previously treated C57BL/6 (*n* = 8). Data represent mean tumor volumes ± SEM. Statistical analysis for **B**–**E** were carried out by 1-way ANOVA with a multiple comparison post test. Statistical significance in **F** and **G** was determined using 2-tailed, unpaired *t* tests. ****P < 0.0001.

Interferon-stimulated genes can both promote and inhibit antitumoral immune responses such that the proimmunogenic effects of T1IFN (e.g., antigen presentation) are counterbalanced by expression of PD-L1 and other immune checkpoints. Therefore, to test the antitumor efficacy of ATM inhibitor and radiotherapy, as well as their ability to sensitize to anti–PD-L1 immune checkpoint blockade, we established mT4 pancreatic tumors in syngeneic C57BL/6 mice that were subsequently treated with AZD1390, radiation, and/or anti–PD-L1 as illustrated (Figure 3A). As anticipated, AZD1390 alone was ineffective in controlling tumor growth or delaying tumor volume doubling (Figure 3, B and C) (21). Radiation alone had modest activity that was similar when administered in combination with AZD1390 or anti–PD-L1 under the radiation conditions used in this study that we previously established to stimulate an innate immune response (20). Consistent with the hypothesis that AZD1390 enhances the radiation-induced antitumoral immune response to sensitize to immunotherapy, the triplet combination of AZD1390, radiation, and anti–PD-L1 significantly inhibited tumor growth and delayed tumor volume doubling with minimal toxicity during therapy, as assessed by weight loss (Figure 3, B and C, and Supplemental Figure 3, A and B). These results were further supported by therapeutic studies of the KPC2/FVB model in which combined therapy with AZD1390, radiation, and anti–PD-L1 significantly inhibited tumor growth, albeit with a greater therapeutic effect of radiation and anti–PD-L1 than observed in mT4 tumors (Supplemental Figure 3, C–E).

To understand if the observed interaction of AZD1390 with radiation and anti–PD-L1 was specific to ATM inhibition, we conducted a similar study with AZD0156, a highly potent ATM inhibitor from the same compound series as AZD1390 (21). Immunocompetent, syngeneic mice with mT4 tumors were treated with AZD0156 in combination with radiation and anti–PD-L1 (Figure 3A). Similar to the results obtained with AZD1390, we found that the addition of AZD0156 to radiation and anti–PD-L1 therapies significantly inhibited mT4 tumor growth and volume doubling (as compared with radiation+anti–PD-L1; Figure 3, D and E).

Effective immunotherapies induce antitumor CD8^+^ T cell memory responses. To test whether the combination of AZD1390, radiation, and anti–PD-L1 generated immune memory, mice cured of their primary tumors were reimplanted with respective syngeneic mT4 or KPC2 tumor cells. As compared with tumor naive mice in which all tumors engrafted and progressed, mice with prior complete response to therapy rejected mT4 or KPC2 tumors for up to 3 months following complete therapeutic response (Figure 3, F and G, and Supplemental Figure 3F). Taken together, these data suggest that combined therapy with an ATM inhibitor and radiation can produce durable antitumoral immune responses to immunotherapy.

### Combined therapy with AZD1390, radiotherapy, and anti–PD-L1 reprograms the pancreatic tumor microenvironment.

**Figure 4:**
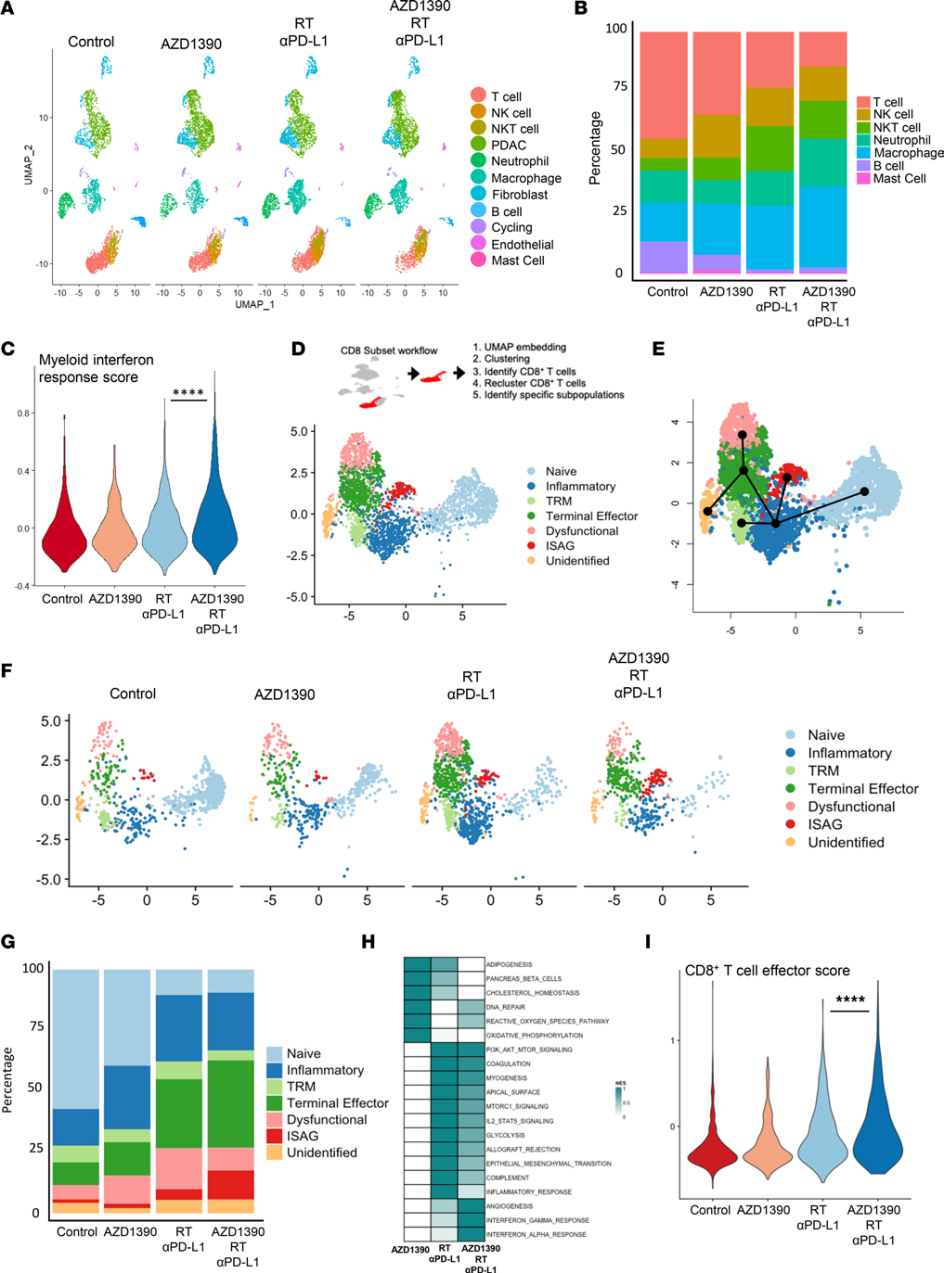
ATM inhibitor, radiotherapy, and anti–PD-L1 treatment alters the tumor immune microenvironment. C57BL/6 mice with mT4 tumors were treated as illustrated (Figure 3A) and harvested for scRNA-Seq analysis at day 10. (**A**) UMAP projection of all the cell clusters from harvested subcutaneous mT4 tumors. (**B**) Frequencies of immune cell clusters from single-cell analysis. (**C**) Myeloid-specific interferon signaling module score by treatment. (**D**) Workflow for subsetting CD8^+^ T cells and UMAP projection of reclustered CD8^+^ T cell populations. (**E**) Trajectory analysis of CD8^+^ T cell clusters. (**F**) UMAP projection of CD8^+^ T cell clusters divided by treatment group. (**G**) Frequency of CD8^+^ T cell clusters in each treatment group. (**H**) Top pathways from gene set enrichment analysis of differentially expressed genes between the control group and each individual treatment. (**I**) CD8^+^ T effector module score from all CD8^+^ T cells divided by treatment. Statistical significance in **C** and **I** was determined using 1-way ANOVA with a multiple comparison post test. ****P < 0.0001.

AZD1390 and radiotherapy enhance anti–PD-L1 efficacy and produce lasting antitumoral responses in pancreatic cancer models. To characterize treatment effects on the pancreatic tumor microenvironment, single-cell RNA-Seq was performed on mT4 tumors following treatment of animals with AZD1390 alone or in combination with radiation plus anti–PD-L1. Using an unbiased approach, we identified 11 immune cell clusters based on the gene expression of well-characterized lineage markers (Figure 4A and Supplemental Figure 4A). All cell clusters were present in all treatment groups (Figure 4B), although the proportion varied (Figure 4B). Myeloid cells are critical mediators of interferon signaling in vivo (35). To understand whether the AZD1390 alone or in combination with radiation plus anti–PD-L1 augmented T1IFN signaling in vivo, we evaluated interferon gene signatures within myeloid cells. We observed that only the combination of AZD1390, radiation, and anti–PD-L1 significantly increased interferon signaling (Figure 4C and Supplemental Figure 4B).

Given the suggested contribution of adaptive immune responses to immunotherapy efficacy (Figure 3), we isolated T cells and reclustered (Supplemental Figure 4C). Based on the expression of *Cd8a*, we performed unsupervised clustering of CD8^+^ T cells, which produced 7 clusters (Figure 4D). Identification was performed on the basis of cluster-specific marker expression: naive T cells with high expression of *Ccr7*, *Lef1*, and *Sell*; tissue-resident inflammatory T cells expressing *Ccl5*, *Gzmk*, and *Itga4* (Cd49a); tissue resident-memory T (TRM) cells expressing *Il7r, Ifitm1, Lgals3, Itgb1, Vim, Crip1, Ccr2,* and *Itgae*, similar to previous reports (36–38); terminal effector T cells expressing both cytotoxic markers (*Ccl4*, *Ifng*, *Prf1*, *Gzmb)* and dysfunction markers *(Pdcd1, Ctla4, Lag3, Havcr2)*; a cluster expressing dysfunction-associated markers *Rgs16, Nr4a2, Cd160, and Tgfb* (39–42)*;* and a cluster termed “ISAG” expressing *Isg15, Stat1, Irf7,* and *Cxcl10* similar to interferon-signaling associated gene T cells that have been previously described (43–45) (Supplemental Figure 4D). To evaluate the relationship between different T cell clusters, we performed pseudotime trajectory analysis with Slingshot. In line with previous studies (46, 47), we observed that naive T cells gave rise to an intermediate inflammatory state and subsequently branched into TRM, interferon-stimulated (ISAG), or terminal effector status (Figure 4E). Terminal effector cells, in turn, became dysfunctional.

There were differences in the frequencies of each T cell cluster across the treatment groups (Figure 4, F and G). Importantly, we noted an expansion in the Ifng/Gzmb^+^ terminal effector populations in response to progressive treatment, with the greatest proportion of Ifng/Gzmb^+^ cells observed in response to combined therapy with AZD1390, radiation, and anti–PD-L1 in association with a corresponding decrease in the naive T cell cluster. We also observed that the combination of AZD1390, radiation, and anti–PD-L1 uniquely reduced the dysfunctional cell cluster and increased the frequency of the ISG cell cluster, which has been associated with rapid adaptive immune responses.

To determine how these treatments might functionally change CD8^+^ T cells in the tumor microenvironment, we performed gene set enrichment analysis (GSEA) of all CD8^+^ T cells and evaluated differentially expressed genes between the control group and each treatment group. Consistent with a role for ATM in DNA damage and metabolism (48), AZD1390 treatment was associated with enrichment of DNA repair pathways and oxidative phosphorylation pathways (Figure 4H). As expected, combined therapy with radiation and anti–PD-L1 was associated with inflammatory response, allograft rejection, and IL2/Stat5 signaling (Figure 4H and Supplemental Figure 4E). To further define changes in the CD8^+^ T cell population that may contribute to the enhanced tumor responses observed by the addition of AZD1390 to radiation and anti–PD-L1, we compared differentially expressed genes between CD8^+^ T cells treated with AZD1390 versus without in combination with radiation and anti–PD-L1. We found that the addition of AZD1390 to radiation and anti–PD-L1 treatment specifically enriched gene signatures associated with interferon responses (type I and II), allograft rejection, and IL2/Stat5 signaling (Supplemental Figure 4F), the latter of which, together with type II interferon (IFN-γ) signaling, may drive a terminal effector differentiation program in CD8^+^ T cells (49). Indeed, T effector module scores using a previously described effector gene signature (50) were highest in the tumors treated with AZD1390, radiation, and anti–PD-L1 (Figure 4I). These data support that there is increased T cell effector function in response to combination treatment with AZD1390, radiation, and anti–PD-L1.

### Augmentation of CD8^+^ T cell immune surveillance in response to combined therapy with AZD1390, radiotherapy, and anti–PD-L1.

**Figure 5:**
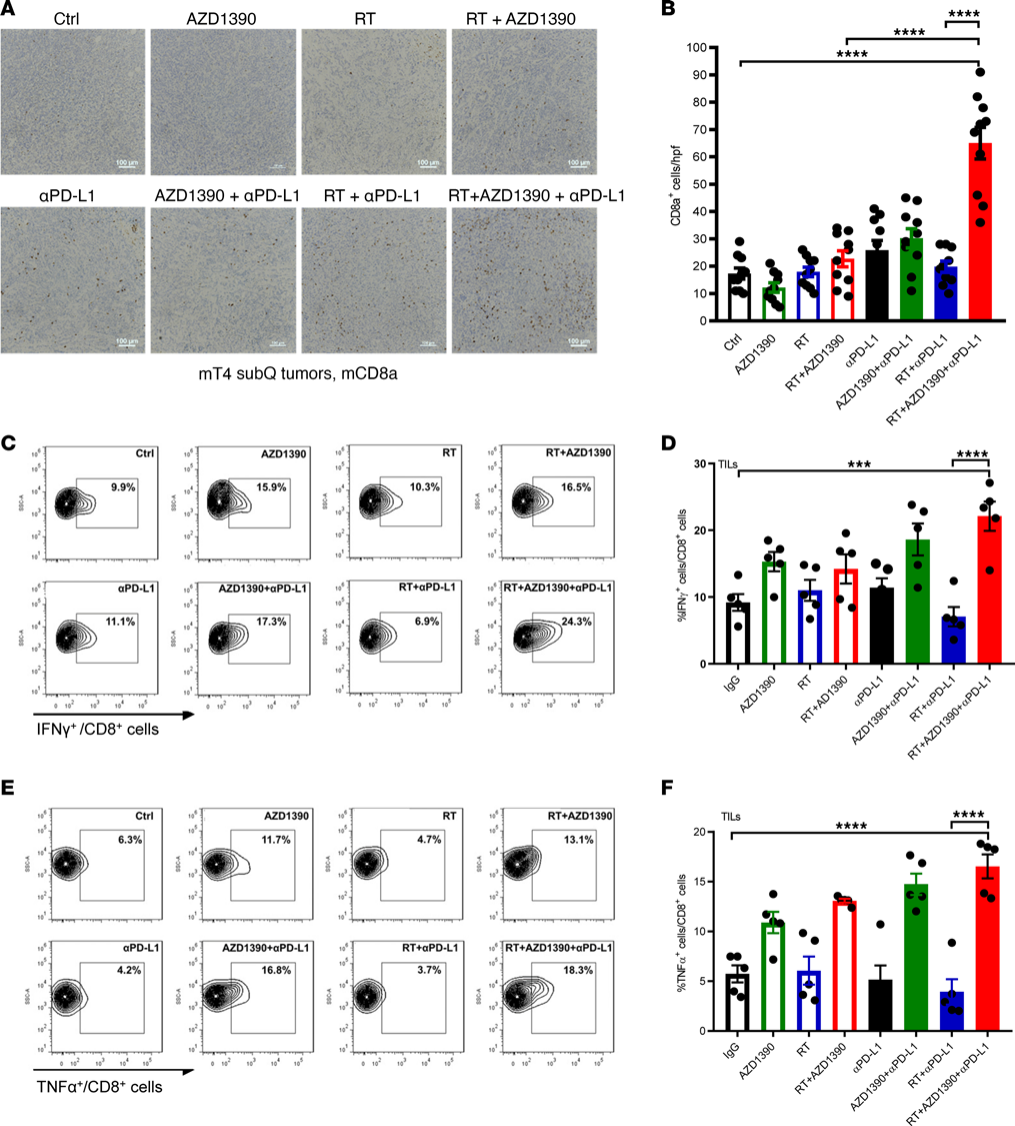
Combined therapy enhances CD8^+^ T cell activity in tumor-infiltrating lymphocytes. (**A** and **B**) C57BL/6 mice with mT4 tumors were treated as illustrated (Figure 3A), harvested at day 10, and stained for CD8a by immunohistochemistry. Data are representative images (**A**) or the mean ± SEM of CD8a^+^ cell number in each bright field (*n* = 10 for each condition (**B**). Scale bars: 100 μm. (**C**–**F**) Flow cytometry analysis of the percentages of tumor IFN-γ^+^CD8^+^ T cells (**C** and **D**) and TNF-α^+^CD8^+^ T cells (**E** and **F**) from mT4 tumors treated as indicated. Data are representative flow cytometry illustrations (**C** and **E**) or the mean ± SEM (**D** and **F**) (*n* = 5 mice/group). Statistical analyses were carried out by 1-way ANOVA with a multiple comparison post test. ***P < 0.001, ****P < 0.0001.

To confirm the expansion and phenotypic changes of CD8^+^ T cells observed by single-cell sequencing, we next immunohistochemically evaluated the intratumoral CD8^+^ T cell population in mT4 tumors in response to treatment. We found that AZD1390 alone or in combination with anti–PD-L1 or radiation did not substantially increase T cell number (Figure 5, A and B). In contrast, combined treatment with AZD1390, radiation, and anti–PD-L1 significantly increased the intratumoral CD8^+^ T cell population compared with doublet therapy combinations of AZD1390 or anti–PD-L1 with radiation. We next characterized the function of the intratumoral CD8^+^ T cells by flow cytometry. Consistent with immunohistochemical staining for CD8^+^ T cells, AZD1390, radiation, and anti–PD-L1 uniquely increased the proportion of CD8^+^ T cells within tumors assessed by flow cytometry analysis (Supplemental Figure 5, A and B). Furthermore, while AZD1390 monotherapy had a modest effect, the combination of AZD1390, radiation, and anti–PD-L1 robustly induced IFN-γ and TNF-α cytokine expression in the CD8^+^ T cells as compared with untreated tumors (Figure 5, C–F). This increase in activated intratumoral CD8^+^ T cells was also accompanied by a decrease in the proportion of exhausted T cells marked by PD-1^+^Tim-3^+^ positivity (Supplemental Figure 5C). The enhanced frequency and cytotoxicity of intratumoral CD8^+^ T cells in the mice treated with the triple combination of AZD1390, radiation, and anti–PD-L1 are consistent with the optimal tumor control observed in Figure 3.

### ATM inhibitor, radiotherapy, and anti–PD-L1 increase systemic tumor control.

**Figure 6:**
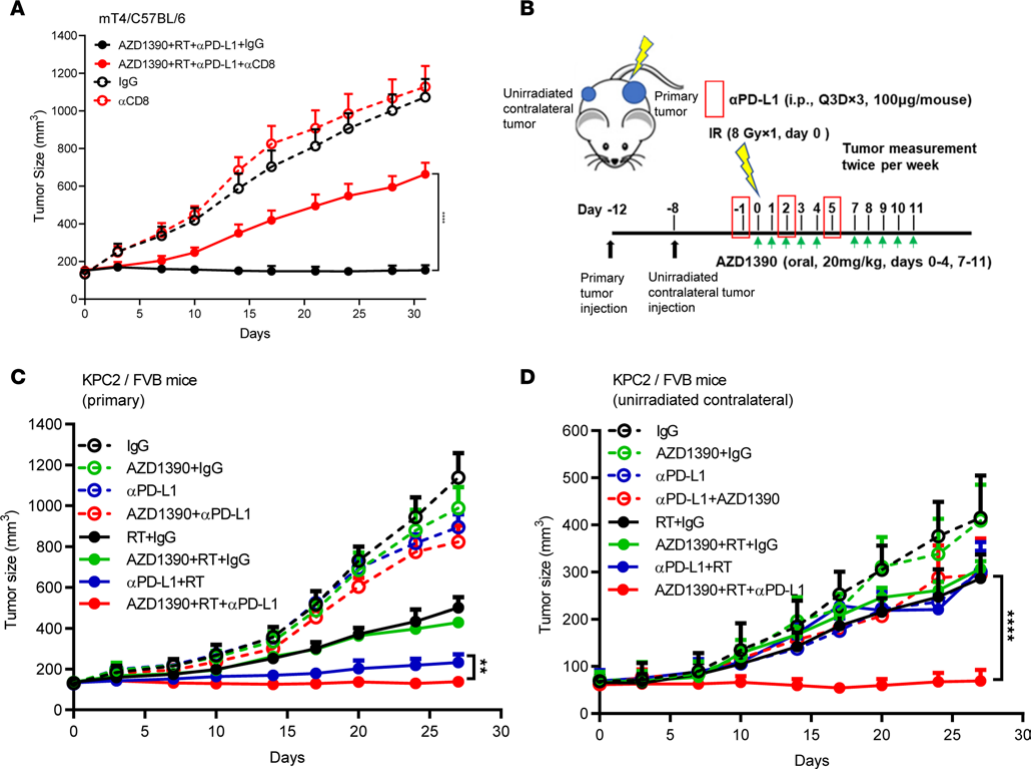
AZD1390, radiotherapy, and anti–PD-L1 generate CD8^+^ T cell–dependent, systemic tumor control. (**A**) mT4 tumor volumes in implanted C57BL/6 mice with or without triple combinational treatment of AZD1390, radiation (RT), and anti–PD-L1 (as illustrated in Figure 3A) in the absence or presence of anti-CD8 antibody (250 μg, administered day –1, 2, 5, and 8). Number of mice per treatment arm = 10 (ctrl), 10 (αCD8), 20 (αPD-L1+AZD1390+RT), and 20 (αCD8+αPD-L1+AZD1390+RT). (**B**) KPC2 and mT4 tumor inoculation and treatment schema showing primary and contralateral tumor implantation at day –12 and –8, respectively. Primary tumors were treated with the same schedule as Figure 3A with the exception of with 3 doses of anti–PD-L1. (**C** and **D**) Tumor growth curves of irradiated tumors (primary, **C**) and unirradiated contralateral tumors (**D**) in KPC2 tumor-bearing mice after the indicated treatments. *N* mice per treatment arm = 6 (ctrl), 6 (AZD1390), 8 (RT), 8 (AZD1390+RT), 8 (αPD-L1), 8 (αPD-L1+AZD1390), 8 (αPD-L1+RT), and 10 (αPD-L1+AZD1390+RT). Data represent the mean ± SEM. Statistical analyses were carried out by 1-way ANOVA with a multiple comparison post test. **P < 0.01, ****P < 0.0001.

Our data suggest that AZD1390, radiation, and anti–PD-L1 enhance the antitumoral CD8^+^ T cell response (Figures 4 and 5). To functionally test the importance of CD8^+^ T cells to combined therapy efficacy, we next established mT4 tumors in mice and treated them with combination therapy both in the presence and absence of CD8-depleting antibodies. Elimination of CD8^+^ T cells substantially reduced AZD1390, radiation, and anti–PD-L1 efficacy (Figure 6A). CD8^+^ T cells can also promote systemic antitumoral immune responses following tumor-targeted delivery of radiation to a local tumor (51, 52). To understand if AZD1390, radiation, and anti–PD-L1 confer systemic antitumor immunity, we implanted bilateral KPC2 tumors in syngeneic mice and treated them with AZD1390, unilateral radiation, and/or anti–PD-L1 (Figure 6B). We found that AZD1390, radiation, and anti–PD-L1, as well as anti–PD-L1 and radiation, induced significant tumor control in the irradiated primary tumors (Figure 6C). Interestingly, only AZD1390, radiation, and anti–PD-L1 resulted in control of the unirradiated contralateral (abscopal) tumors (Figure 6D). To confirm these findings, we repeated this experiment in the independent mT4/C57BL/6 model. Similarly, AZD1390, radiation, and anti–PD-L1 were most effective in controlling the irradiated tumor (Supplemental Figure 6A) and were also the only treatment that resulted in control of contralateral tumors outside of the radiation field (Supplemental Figure 6B).

## Discussion

In this study, we showed that the ATM inhibitor AZD1390 in combination with radiation can induce T1IFN-mediated antitumoral immune responses in otherwise immunologically suppressed pancreatic cancer. We found that enhanced T1IFN expression by ATM inhibitor in combination with radiation was mediated by the POLIII/RIG-I/MAVS signaling pathway, independent of the canonical cGAS/STING pathway, suggesting the potential dysregulation of cGAS/STING signaling in pancreatic cancer. Regarding therapeutic potential, our data in two independent syngeneic mouse models of pancreatic cancer showed the ability of AZD1390 (and a structurally related compound AZD0156) to sensitize tumors to PD-L1 immune checkpoint inhibition when given in combination with radiotherapy. Single-cell RNA-Seq analysis suggested an enhanced CD8^+^ T cell–mediated adaptive immune response following combination therapy with AZD1390, radiation, and anti–PD-L1. The involvement of CD8^+^ T cells in antitumor immune responses to combined treatment was further supported by corresponding increases in both the number and activation status of intratumoral CD8^+^ T cells in response to treatment as well as their requirement for in vivo therapeutic efficacy and sufficiency to promote therapeutic response. Taken together, these results suggest that AZD1390 in combination with radiation enhances the CD8^+^ T cell–mediated adaptive immune response to sensitize pancreatic cancer to immunotherapy.

Genetic silencing of ATM in tumor cells promotes radiation-induced T1IFN-mediated antitumoral immune responses (20). Consistent with this finding, our data illustrate that pharmacologic inhibition of ATM by AZD1390 (or AZD0156) also enhances radiation-induced T1IFN responses in pancreatic cancer cells. Unlike genetic manipulation in tumor cells, however, systemic therapies like AZD1390 also inhibit ATM in the tumor immune microenvironment. As such, ATM activity is likely inhibited in other cellular immune compartments. Consistent with a possible direct effect of ATM in CD8^+^ T cells, we noted a robust of effect of AZD1390 monotherapy on the activation of CD8^+^ T cells (marked by IFN-γ and TNF-α expression) independent of additional therapies (Figure 5). This finding is supported by a direct role of ATM in regulating senescence of CD8^+^ T cells (53). Increases in activated, intratumoral CD8^+^ T cells following radiotherapy have been attributed to radiation effects on tumor-resident T cells (54). It is possible that the observed increase in intratumoral CD8^+^ T cells we observed following combined therapy is due to either expansion of resident and/or infiltration of CD8^+^ T cells from the periphery. Collectively, these data raise the possibility that ATM, similar to ATR (55, 56), may have a direct role in regulating tumoral CD8^+^ T cells.

Our current and prior studies demonstrate that inhibition or silencing of ATM enhances PD-L1 expression following radiation (20). These findings contrast with an earlier report describing a requirement for ATM in PD-L1 expression following radiation (57). Different model systems as well as ATM intervention strategies may account for this difference; however, we note that the contrasting study utilized a less potent, earlier generation ATM inhibitor (KU55933) (58) in mechanistic studies limited to the U2OS cell line. This study highlighted an ATR/CHK1-driven mechanism of PD-L1 induction following radiation, with ATM serving as an upstream factor to initiate end resection at double-strand breaks, thereby leading to the recruitment of ATR/CHK1 to resected single-stranded DNA and ultimately PD-L1 expression. The requirement for ATR/CHK1 signaling is consistent with work from Vendetti and colleagues (55), which demonstrated that ATR inhibition blocks radiation-induced PD-L1. While it is possible that there is a specific function for ATM in mediating ATR/CHK1 signaling leading to PD-L1 induction, our study demonstrates from a broad perspective, including potent ATM inhibitors, multiple cell lines, and functional studies of CD8^+^ T cells that ATM inhibition enhances PD-L1 expression in pancreatic cancer treated with radiation, likely surpassing any potential negative regulation mediated by ATM/ATR/CHK1 signaling.

While microsatellite instability is a useful predictor of immunotherapy efficacy in some cancers, nearly all pancreatic cancers are microsatellite stable. Radiation has been combined with immunotherapy to overcome resistance in microsatellite stable pancreatic cancer but with modest success (14). Thus, conceptually this study aimed to further potentiate the immunogenic effects of radiation by inhibition of the ATM-mediated DNA damage response. While a patient selection strategy will be the focus of future investigation, consideration of the genetic characteristics known to confer responses to the combination of ATM inhibitor and radiation are relevant. Specifically, mutations of TP53, present in the vast majority of pancreatic cancer (59), confer increased susceptibility to radiosensitization by ATM inhibition/loss that is attributed to differential regulation of apoptosis in P53 mutant versus wild-type tumors (60, 61). Furthermore, P53 mutation is associated with the ability of other DNA damage response inhibitors, including those targeting DNA-PK and ATR, to enhance radiation-induced T1IFN antitumor immune responses (16, 34). Taken together, these data support the potential for DNA damage response inhibitors like AZD1390 to selectively enhance the radiation-induced antitumoral immune response and sensitivity to immunotherapy in P53 mutant pancreatic cancer.

ATM, together with ATR and DNA-PK, catalytic subunit, constitutes the phosphatidylinositol 3-kinase-like kinase family of proteins, which, through phosphorylation of downstream effector proteins as well as autophosphorylation, mediate the cellular response to DNA damage (17). While this study describes the ability of a therapeutic agent targeting ATM to enhance the radiation-induced antitumoral immune response, it is supported by studies demonstrating that clinical candidate DNA-PK inhibitors (peposertib, ceralasertib) enhance T1IFN-dependent antitumoral immune responses (12, 16, 62). Furthermore, inhibitors of ATR such as ceralasertib were among the first DNA damage response inhibitors shown to enhance antitumoral immune responses by modulation of both the T1IFN response in tumor cells as well as direct modulation of CD8^+^ T cells dysfunction (34, 55). These data together with our prior work characterizing a role for ATM in restraining the innate immune response to radiation support an overall role for the DNA damage response in innate immune signaling and the antitumoral immune response following radiotherapy (20). While these findings collectively support that inhibitors of the DNA damage response enhance the radiation-induced innate immune response in tumor cells, it is likely, given the differential abilities of ATM, ATR, and DNA-PK to modulate DNA repair, replication stress, and cell cycle checkpoints, that therapies targeting these proteins will also vary in terms of their ability to induce antitumoral immunity. Our data herein demonstrate compelling efficacy of ATM inhibitor in enhancing radiation-induced antitumor immune responses in pancreatic cancer.

Overall, this study highlights the potential for the first-in-class ATM inhibitor AZD1390 to enhance radiation-induced antitumoral immune responses and immunotherapy efficacy in pancreatic cancer. This finding has implications for the next generation of clinical trials, given that current phase I studies are designed to establish a safe dose of AZD1390 with radiation to which immune checkpoint inhibitors could be integrated. Translation of these preclinical studies to immunologically suppressed tumors such as pancreatic cancer with the goal of maximizing both the innate and adaptive antitumoral immune response to improve both local and systemic disease therapy in patients is a critical next step.

## Methods

### Sex as a biological variable.

Six- to eight-week-old female wild-type FVB mice (Envigo), or C57BL/6 mice (The Jackson Laboratory) were used. Sex was not considered as a biological variable in these studies as the incidence and outcome of human pancreatic cancer is similar for both sexes.

### Reagents and cell lines.

The ATM inhibitors AZD0156 and AZD1390 were synthesized and provided by AstraZeneca. The RNA POLIII inhibitor ML-60128 was manufactured by Focus Biomolecules. The PE-labeled anti-human PD-L1 (MIH2) and anti-mouse PD-L1 (10F.9G2) antibodies and their relative isotypes were obtained from Biolegend. The mouse PD-L1 blocking antibody (10F.9G2, BE0101), CD8α blocking antibody (2.43, BP0061), and IgG1 isotype control (LTF-2, BE0090) were purchased from BioXCell. The CD8a (D4W2Z, 98941) antibody for immunohistochemical staining was purchased from Cell Signaling Technology. AZD0156 and AZD1390 was dissolved in dimethyl sulfoxide (MilliporeSigma) for in vitro experiments and stored in aliquots at –20°C. The human pancreatic cancer cell line Panc1 and mouse pancreatic cancer cell line mT4 (C57BL/6 background) were grown in DMEM with 10% FBS (Hyclone), while the mouse pancreatic cancer cell line KPC2 (also known as 65.671, FVB/NJ background) was grown in RPMI-1640 with 10% FBS. The Panc1 cell line was obtained from ATCC. mT4 and KPC2 cells were obtained from David Tuveson (Cold Spring Harbor Laboratory, Cold Spring Harbor, New York, USA) (63) and Pasca di Magliano (University of Michigan) (64), respectively. The establishment and characterization of Panc1 cGAS-, STING-, or TBK1-KO cells; Panc1 POL3A-, MAVS-, or RIG-I–knockdown cells; and KPC2 cGas-, Sting-, or Tbk1-KO cells was described previously (12). All cell lines were tested for Mycoplasma every 3 months and authenticated by short-tandem repeat profiling.

### Irradiation.

Cells were irradiated at a dose rate of approximately 2 Gy/min with a 225-kilovolt beam energy by using a Philips RT250 (Kimtron Medical) at the Experimental Irradiation Shared Resource of University of Michigan Rogel Comprehensive Cancer Center. Dosimetry was performed using an ionization chamber connected to an electrometer system that is directly traceable to a National Institute of Standards and Technology calibration. For irradiation of mouse flank tumors, isoflurane was used to anesthetize tumor-bearing animals, and tumors were set at the center of a 2.4 cm circular aperture in a custom lead holder in order to shield the rest of the mouse from radiation.

### IFNβ1–GFP reporter assay.

The pLKO.1-hygro-IFNβ–GFP reporter was a gift from Roger A. Greenberg (University of Pennsylvania, Philadelphia, Pennsylvania, USA) (5). Panc1 cells were transfected with the IFN-β–GFP reporter, and stable transfectants were selected with 50 μg/mL hygromycin. The established Panc1-IFNβ–GFP reporter cells were treated with AZD1390 or AZD0156 (1 hour before radiation) and/or radiation and harvested after 3 days upon treatment. GFP expression levels of reporter cells with various treatment were measured by flow cytometry (BD Biosciences). The change in median fluorescence intensity (MFI) for indicated treatments was obtained by subtraction of background GFP levels. Detailed methods can be found in our previous studies (12, 20).

### Quantitative RT-PCR.

RNA was isolated with the RNeasy Mini Kit (Qiagen) and DNase digestion (Qiagen) from cells with indicated treatment. RNA concentration and purity were measured using a Nanodrop spectrophotometer (Thermo Fisher Scientific). cDNA was reverse transcribed using SuperScript III First-strand Synthesis System for RT-PCR (Invitrogen, 12574026) (65). Relative indicated gene expression levels were determined by quantitative PCR (qPCR) using Fast SYBR Green Master Mix (Thermo Fisher Scientific, A46109) on a StepOnePlus Real-Time PCR System (Thermo Fisher Scientific) and fold change (ΔΔC_t_) method normalized to *β-actin*. The following qPCR primers were used: human *IFNβ* (forward), 5′-ATGACCAACAAGTGTCTCCTCC-3′, human *IFNβ* (reverse), 5′-GCTCATGGAAAGAGCTGTAGTG-3′; human *CXCL9* (forward), 5′-GTGGTGTTCTTTTCCTCTTGGG-3′, human *CXCL9* (reverse), 5′-ACAGCGACCCTTTCTCACTAC-3′; human *CXCL10* (forward), 5′-CTCCAGTCTCAGCACCATGA-3′, human *CXCL10* (reverse), 5′-GCTCCCCTCTGGTTTTAAGG-3′; mouse *Cxcl9* (forward), 5′-CCTAGTGATAAGGAATGCACGATG-3’, mouse *Cxcl9* (reverse), 5′-CTAGGCAGGTTTGATCTCCGTTC-3′; and mouse *Cxcl10* (forward), 5′-CCTGCCCACGTGTTGAGAT-3′, mouse *Cxcl10* (reverse), 5′-TGATGGTCTTAGATTCCGGATTC-3′.

### Immunofluorescence.

Panc1 cells were seeded onto coverslips in 12-well plates and treated with AZD0156 or AZD1390 and/or radiation. After 3 days, coverslips were washed with cold PBS and mounted with a drop (~10 μL) of ProLong Gold Antifade with DAPI (Invitrogen, P36935). Images were captured using an Olympus IX71 FluoView confocal microscope (Olympus America) with a ×60 oil objective and Nikon NIS-Elements software. Images were then prepared using Fiji (NIH) software by equivalently adjusting only for brightness and contrast. Micronucleated cells were counted manually for each field and classified by distinct staining by DAPI of structures outside of main nuclei. The percentage of micronucleated cells was determined by micronuclei-positive cells of total cells within the field. At least 200 cells from each treatment condition were evaluated.

### Flow cytometry.

To analyze cell surface PD-L1 expression of Panc1 and KPC2 cells with indicated treatment, cells were trypsinized to generate single-cell suspension in 50–200 μL cell staining buffer (420201, BioLegend) based on cell numbers. Cells were then incubated with PE-conjugated anti-human PD-L1 antibody or anti-mouse PE-labeled PD-L1 antibody for 1 hour at room temperature in the dark. Stained cells were washed in the staining buffer and analyzed by flow cytometry (BD Biosciences). The PD-L1 expression levels on the cell surface were analyzed in FlowJo 7.6 software. Background-corrected PD-L1 MFI (i.e., PD-L1 MFI minus isotype control MFI for each treatment condition) was calculated and analyzed. dsRNA (76651, Cell Signaling Technologies) was detected 3 days after treatment. Levels of all fluorophores were analyzed via flow cytometry (BD LSR Fortessa) and analyzed using FlowJo 7.6 software.

### Western blot analysis.

For whole-cell protein extracts, cells were collected and immediately homogenized in RIPA buffer (50 mM Tris [pH 7.5], 1% NP40, 0.5% SDS, 150 mM NaCl, 1 mM EDTA [pH 8.0]), supplemented with protease and phosphatase inhibitors (Roche). Protein concentration was determined using Bradford protein assay (Bio-Rad). Equal amounts of cell lysates were denaturated in 2× Laemmli buffer for 10 minutes at 100°C. Samples were then resolved by SDS-PAGE and transferred to PVDF membranes (0.2 μm). The antibodies against POLR3A (1:1,000, D5Y2D, 12825), RIG-I (1:1,000, D14G6, 3743), MAVS (1:1,000, D5A9E, 24930), phosphor-TBK1 (1:1,000, D52C2, 5483), TBK1 (1:1,000, 3013), cGAS (1:1,000, D1D3G, 15102), STING (1:1,000, D2P2F, 13647), phosphor-ATM (1:1,000, D6H9, 5883), HA (1:2000, C29F4, 3724), and GAPDH (1:5,000, D16H11, 5174) were purchased from Cell Signaling Technologies.

### In vivo mouse models.

KPC2 and mT4 pancreatic cancer cells (1 × 10^6^) were subcutaneously injected to the left and right flanks of FVB (KPC2) or C57BL/6 (mT4) mice. For primary tumor irradiation with exclusion of the contralateral tumor, mice received injections of KPC2 or mT4 cells on one side (1 × 10^6^) and subsequently received contralateral injections of the same tumor line 4 days thereafter. AZD1390 was prepared as a suspension of 0.5% hydroxypropyl methylcellulose + 0.1% (v/v) Tween 80 in ddH_2_O. PD-L1 blocking antibody or IgG1 isotype control was given intraperitoneally 100 μg/mouse every 3 days starting at day –1 upon AZD1390 administration and radiation when tumors reached approximately 150 mm^3^. AZD1390 was given by oral gavage 1 hour before a single fraction of radiation (8 Gy). Tumor diameters (length [*a*]; width [*b*]) were measured using calipers twice per week. Tumor volume (T_V_) was calculated according to the equation T_V_ = π/6 (*a* × *b*^2^) and plotted as tumor growth curves and doubling time.

### Immunohistochemistry.

Pancreatic tumor tissues were fixed in 10% formalin and embedded in paraffin. Five μm thick sections were cut and baked for 60 minutes at 60°C, and subsequently deparaffinized in xylene and rehydrated in water by decreasing strengths of alcohol. The slides were then subjected to antigen retrieval in 1X AR6 buffer (PerkinElmer) using microwave treatment. CD8 IHC staining (D4W2Z, 98941) was performed using EnVision G|2 Doublestain System (Agilent) as previously described (12). Sections were left to air dry and mounted with permanent mounting medium. Bright-field images were acquired with an Olympus BX-51 microscope, Olympus DP71 digital camera, and DP Controller software. The number of CD8^+^ cells per field were calculated manually and plotted.

### Flow cytometry analysis of intratumoral T cells.

Tumor tissues (around 0.5 g) from 5 or more mice in each arm were used for flow analysis. Tumor tissues were cut into small pieces and transferred in 50 mL tubes containing 10 mL digestion buffer (1 mg/mL collagenase I, 1 mg/mL collagenase IV, and 0.15 mg/mL DNase I in RPMI-1640 medium) and then incubated in a 37°C shaker (180 rpm) for 20–30 minutes. Digested tumor samples were filtered and smashed in 70 μm cell strainer, and the cell strainers were washed several times with FACS buffer (2% FBS in PBS). Cell suspensions were harvested in 50 mL tubes, spun at 300*g* for 5 minutes, and then resuspended with 10 mL FACS buffer. Cell suspensions were loaded on the top of 10 mL Ficoll and were spun at 800*g* for 20 minutes without brake and mild acceleration. The cells within intermediate layer were collected into a new 50 mL tubes and were washed once with FACS buffer and spun at 300*g* for 5 minutes. The cell pellet was used for the following treatment and flow analysis. For cytokine expression analysis, cells were treated with PMA (5 ng/mL, Sigma, P1585), Ionomycin (500 ng/mL, Sigma, I9657), GolgiPlug (1:1,000, BD Biosciences, 555029) and GolgiStop (1:1,000, BD Biosciences, 554724) protein transport inhibitors in incubator for 4 hours at 37˚C. After treatment, cells were stained in FACS buffer (2% FBS in PBS) for surface markers as well as cell viability dye. Then, cells were fixed/permeabilized by using a fixation/permeabilization kit (eBiosciences, 00-5123-43, 00-5223-56) and stained with the cytokines or intercellular markers shown below. The counting beads were used for quantification. A BD Fortessa instrument was used for flow cytometry and FlowJo software for data analysis. Antibodies used include anti-mouse CD90.2 FITC (BD Biosciences, 553004), anti-mouse CD4 (BioLegend, RM4-5), APC-eFluor 780 (eBioscience, 50-112-8895), anti-mouse CD8a APC-R700 (eBioscience, 56-0081-82), anti-mouse IFN-γ (XMG1.2) BV786 (BD Biosciences, 563773), anti-mouse TNF-α PE-Cy7 (eBioscience, 25-7321-82), anti-mouse Tim-3 (RMT3-23) BV605 (BioLegend, 119721), and anti-mouse PD-1 (29F.1A12) PE (BioLegend, 135205).

### Single-cell RNA-Seq.

Subcutaneous tumors (6–8 tumors in each group) were harvested, minced, and digested in a collagenase digest buffer (1 mg/mL collagenase I, 1 mg/mL collagenase IV, and 0.15 mg/mL DNase I in RPMI-1640 medium) for 30 minutes at 37°C. This tissue digest was filtered using a 40 μm mesh filter, and the collagenase was quenched by washing with PBS with 2% FBS. The resulting cells were counted and depleted of dying cells twice using the Dead Cell Removal kit from Miltenyi per the manufacturer’s protocol. To even the number of immune cells and tumor cells for sequencing, a magnetic CD45 isolation kit (Miltenyi) was used. CD45^+^ and CD45^–^ cells were then counted and resuspended at a concentration of approximately 1,000 cells/μL. In equal amounts, the CD45^+^ and CD45^-^ cells from each sample were mixed back together. Single-cell suspensions were subjected to final cell counting on the Luna Fx7 Automated Cell Counter (LogosBio) and diluted to a concentration of 700–1,000 cells/μL. Only samples with more than 85% viability were processed for further sequencing. Single-cell sequencing was performed at the University of Michigan Advanced Genomics Core Research Facility. Single-cell 3′ library generation was performed on the 10x Genomics Chromium Controller following the manufacturer’s protocol for the 3′ v3.1 chemistry with NextGEM Chip G reagents (10X Genomics). Final library quality was assessed using the LabChip GXII HT (PerkinElmer), and libraries were quantified by Qubit (Thermo Fisher). Pooled libraries were subjected to 150 bp paired-end sequencing according to the manufacturer’s protocol (Illumina, NovaSeq 6000). Bcl2fastq2 Conversion Software (Illumina) was used to generate demultiplexed Fastq files, and the CellRanger v7 Pipeline (10X Genomics) was used to align reads and generate count matrices.

### Bioinformatics analysis of single-cell sequencing data.

A total of approximately 100 million reads were generated from the 10X Genomics sequencing analysis for each of the replicates. The sequencing data were first preprocessed using the 10X Genomics software Cell Ranger (10x Genomics Inc.); this step includes alignment against mm10 genome. The Cell ranger summary indicated 94% of the input reads as aligned with approximately 2,600 median genes/cell. Further downstream analysis steps were performed using the Seurat R package (Satija lab; v4). We filtered out cells with less than 200 genes per cell and with more than 10% mitochondrial read content. The downstream analysis steps for each sample type include normalization, identification of highly variable genes across the single cells, scaling based on number of UMI, dimensionality reduction (PCA and UMAP), unsupervised clustering, and the discovery of differentially expressed cell-type-specific markers. After clusters were identified, the T cell cluster was subset and further subclustered into CD4^+^, CD8^+^, and NK T cells. The gene signatures from CD8^+^ T cell clusters were compared between the different treatment groups by GSEA analysis. Further, the CD8^+^ T cell subset was again subclustered to identify memory, tissue-resident, progenitor dysfunction, and dysfunctional CD8^+^ T cells based on cluster-specific markers. CD8^+^ T^+^ cell pseudotime trajectory was analyzed using the Slingshot package from Bioconductor (66).

### Gene signature GSEA analysis.

GSEA analysis was done in R. Fold changes for all genes were calculated with the FoldChange function in Seurat between treatments. Genes that had an average log_2_ fold change (avg_log_2_FC) were filtered out before GSEA. Hallmark pathways from the MSigDB package, which contains pathways from the Molecular Signatures Database (MSigDB; https://www.gsea-msigdb.org/gsea/msigdb), were used to run GSEA using the clusterProfiler package. Pathway heatmaps were created using normalized enrichment scores with the ComplexHeatmap package. GSEA enrichment plots were created with the clusterProfiler package.

### Statistics.

Unless otherwise stated, all data are presented as mean ± SEM. When assessing statistical significance between 2 treatment groups, continuous variables were analyzed using the unpaired 2-tailed Student’s *t* test and Mann-Whitney test for normally and nonnormally distributed data, respectively. In cases of more than 2 groups, 1-way ANOVA with the Tukey’s post-comparison test or Kruskal-Wallis analysis was used. Differences in the time taken to reach 2 times the tumor volume at the start of treatment (i.e., tumor volume doubling time) were examined using the log-rank test. *P* values of less than 0.05 were considered statistically significant and are denoted in the figures. All tests were 2-sided. All statistical analyses were performed using GraphPad Prism 8 statistical software.

### Study approval.

All animal experiments complied with ethical regulations and were approved by University of Michigan Institutional Animal Care and Use Committee (IACUC, PRO00010609). The study was conducted in accordance with local legislation and institutional requirements.

### Data availability.

Single-cell RNA-Seq data were deposited in the NCBI’s Gene Expression Omnibus database (GSE254624). Values for all data points in graphs are reported in the Supporting Data Values file.

## Author contributions

MDG and MAM conceived and designed the study. QZ, LJ, and WW performed most of the experiments. VMV performed qPCR and flow cytometry in mT4 cells. KMJ performed dsRNA flow cytometry. EAH, ANP, and ZW performed some mT4 and KPC2 mouse work. LAP, VMV, and JDP performed cell cycle analysis. AKH, ST, and AR performed single-cell RNA-Seq analysis. QZ, MDG, and MAM wrote the manuscript. QZ, DRW, AR, VS, TSL, MDG, and MAM read and edited the manuscript. QZ and LJ are listed as co–first authors. QZ appears first because QZ established the in vitro and in vivo models, did most of the experiments, and wrote the manuscript.

## Supplementary Material

Supplemental data

Unedited blot and gel images

Supporting data values

## Version 1 02/20/2024

In-Press Preview

## Version 2 03/22/2024

Electronic publication
