# Supplementary material for: Potentiating the radiation-induced type I interferon antitumoral immune response by ATM inhibition in pancreatic cancer
Source: JCI Insight. 2024 Feb 20;9(6):e168824. doi: 10.1172/jci.insight.168824 (PMC11063931; doi:10.1172/jci.insight.168824)
Supplement: Supplemental data [file jciinsight-9-168824-s106.pdf]

Figure S1

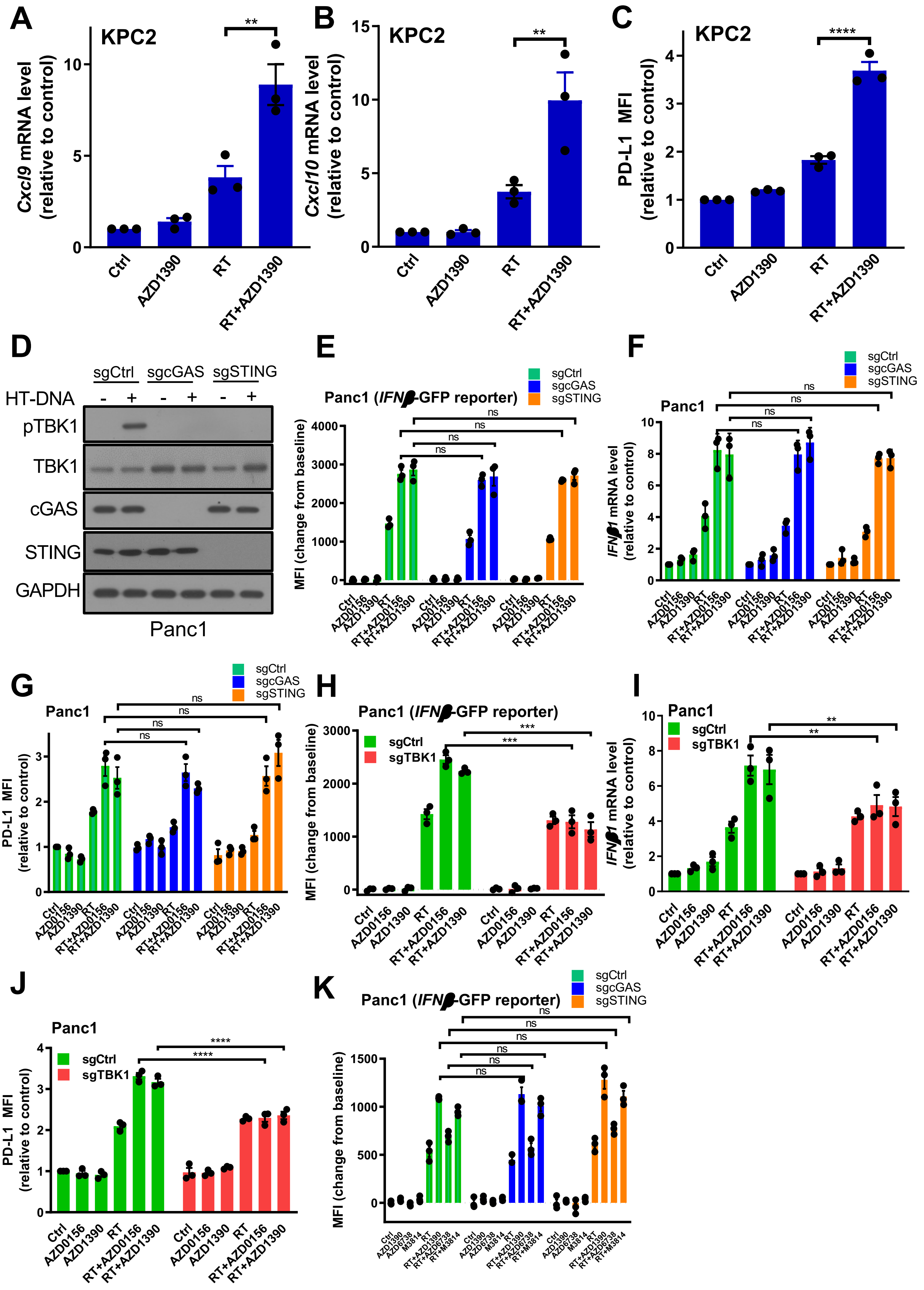

A
KPC2
Cxcl9 mRNA level (relative to control)
Ctrl
AZD1390
RT
RT+AZD1390
B
KPC2
Cxcl10 mRNA level (relative to control)
C
KPC2
PD-L1 MFI (relative to control)
D
sgCtrl
sgcGAS
sgSTING
HT-DNA
pTBK1
TBK1
cGAS
STING
GAPDH
Panc1
E
Panc1 (IFNβ-GFP reporter)
MFI (change from baseline)
AZD0156
F
Panc1
IFNβ1 mRNA level (relative to control)
G
Panc1
PD-L1 MFI (relative to control)
H
Panc1 (IFNβ-GFP reporter)
sgCtrl
sgTBK1
I
Panc1
J
Panc1
K
Panc1 (IFNβ-GFP reporter)
AZD6738
M3814
RT+AZD6738
RT+M3814

Figure S2

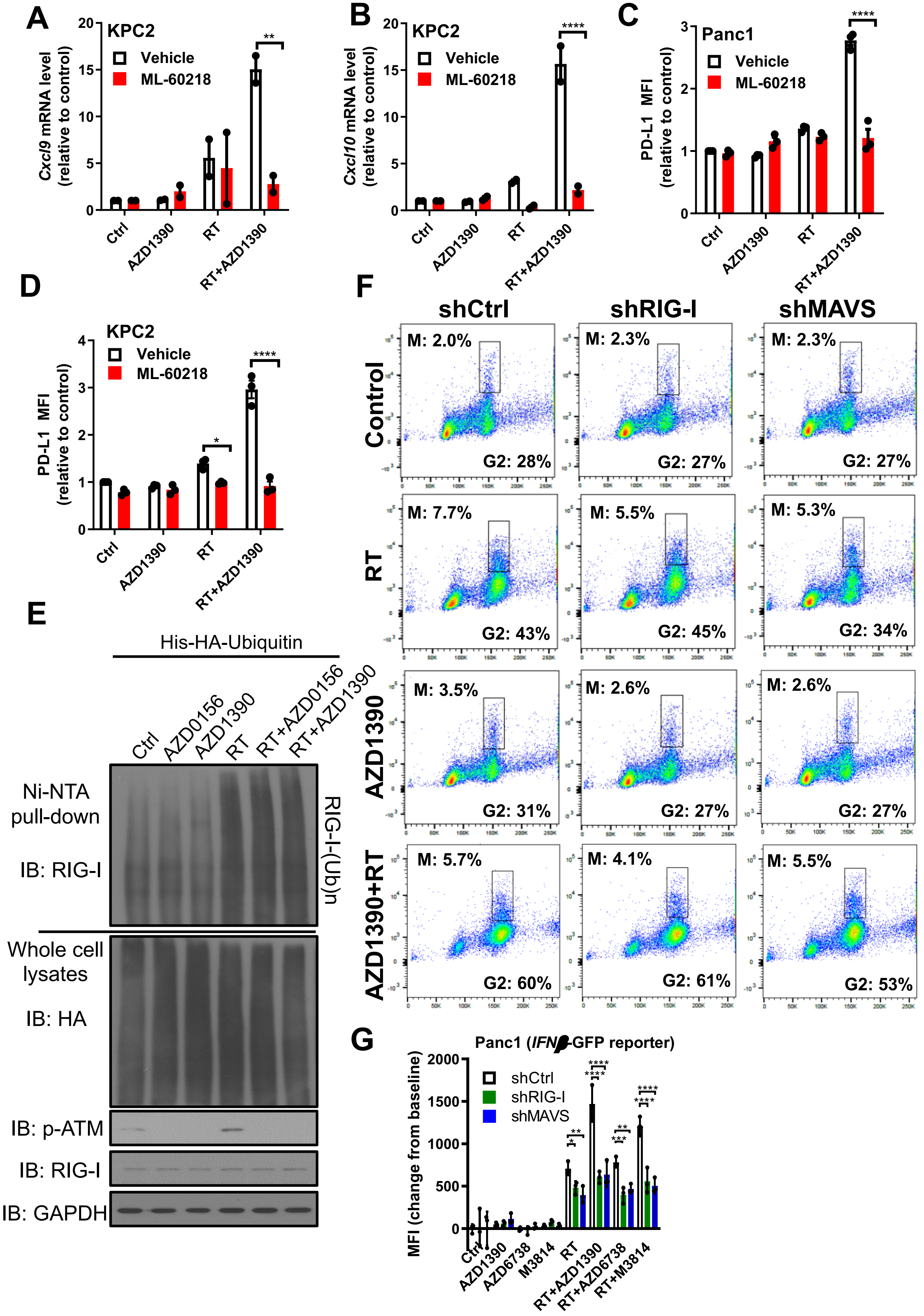

A
KPC2
Vehicle
ML-60218
Cxcl9 mRNA level (relative to control)
0
5
10
15
20
**
Ctrl
AZD1390
RT
RT+AZD1390
B
KPC2
Vehicle
ML-60218
Cxcl10 mRNA level (relative to control)
0
5
10
15
20
****
Ctrl
AZD1390
RT
RT+AZD1390
C
Panc1
Vehicle
ML-60218
PD-L1 MFI (relative to control)
0
1
2
3
****
Ctrl
AZD1390
RT
RT+AZD1390
D
KPC2
Vehicle
ML-60218
PD-L1 MFI (relative to control)
0
1
2
3
4
*
****
Ctrl
AZD1390
RT
RT+AZD1390
E
His-HA-Ubiquitin
Ctrl
AZD0156
AZD1390
RT
RT+AZD0156
RT+AZD1390
Ni-NTA pull-down
IB: RIG-I
RIG-I-(Ub)n
Whole cell lysates
IB: HA
IB: p-ATM
IB: RIG-I
IB: GAPDH
F
shCtrl
shRIG-I
shMAVS
Control
M: 2.0%
G2: 28%
M: 2.3%
G2: 27%
M: 2.3%
G2: 27%
RT
M: 7.7%
G2: 43%
M: 5.5%
G2: 45%
M: 5.3%
G2: 34%
AZD1390
M: 3.5%
G2: 31%
M: 2.6%
G2: 27%
M: 2.6%
G2: 27%
AZD1390+RT
M: 5.7%
G2: 60%
M: 4.1%
G2: 61%
M: 5.5%
G2: 53%
G
Panc1 (IFNβ-GFP reporter)
shCtrl
shRIG-I
shMAVS
MFI (change from baseline)
0
500
1000
1500
2000
*
**
****
****
***
**
****
****
Ctrl
AZD1390
AZD6738
M3814
RT
RT+AZD1390
RT+AZD6738
RT+M3814

Figure S3

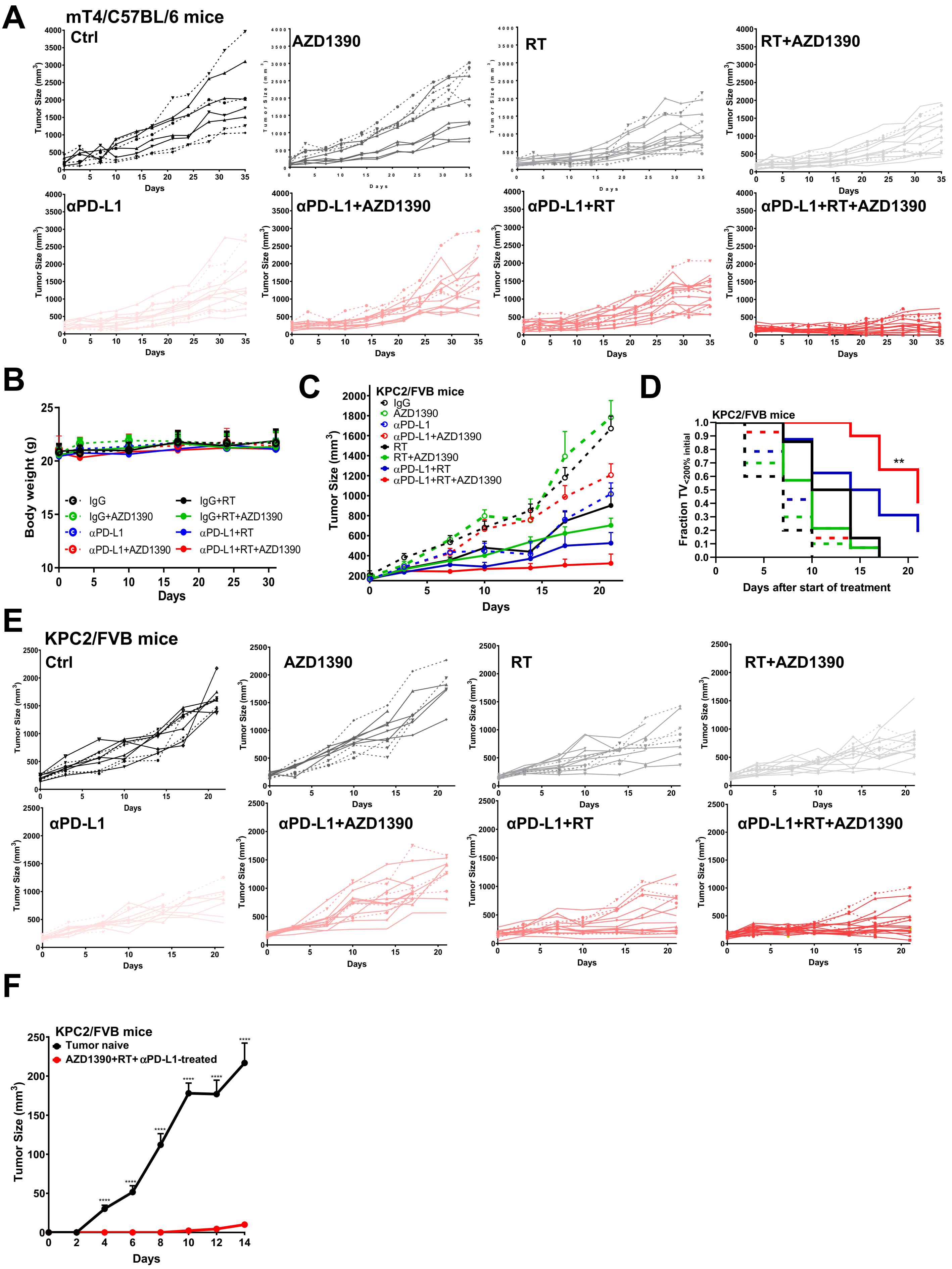
A
mT4/C57BL/6 mice
Ctrl
AZD1390
RT
RT+AZD1390
αPD-L1
αPD-L1+AZD1390
αPD-L1+RT
αPD-L1+RT+AZD1390
Tumor Size (mm3)
Days
B
Body weight (g)
IgG
IgG+AZD1390
αPD-L1
αPD-L1+AZD1390
IgG+RT
IgG+RT+AZD1390
αPD-L1+RT
αPD-L1+RT+AZD1390
Days
C
KPC2/FVB mice
IgG
AZD1390
αPD-L1
αPD-L1+AZD1390
RT
RT+AZD1390
αPD-L1+RT
αPD-L1+RT+AZD1390
Tumor Size (mm3)
Days
D
KPC2/FVB mice
**
Fraction TV<200% initial
Days after start of treatment
E
KPC2/FVB mice
Ctrl
AZD1390
RT
RT+AZD1390
αPD-L1
αPD-L1+AZD1390
αPD-L1+RT
αPD-L1+RT+AZD1390
Tumor Size (mm3)
Days
F
KPC2/FVB mice
Tumor naive
AZD1390+RT+αPD-L1-treated
****
Tumor Size (mm3)
Days

Figure S4

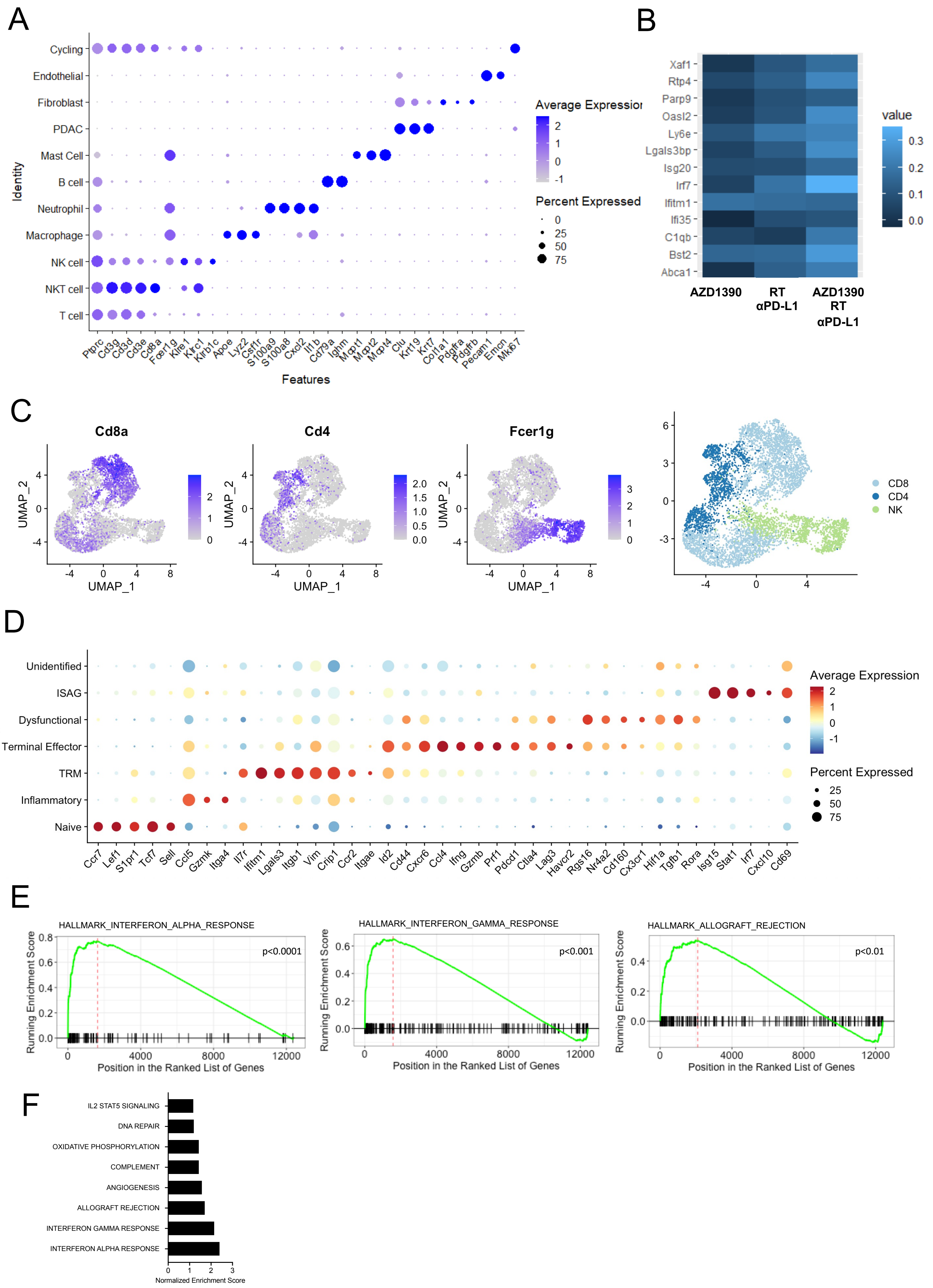

A
B
C
D
E
F
Cycling
Endothelial
Fibroblast
PDAC
Mast Cell
B cell
Neutrophil
Macrophage
NK cell
NKT cell
T cell
Identity
Features
Average Expression
Percent Expressed
value
AZD1390
RT αPD-L1
AZD1390 RT αPD-L1
Cd8a
Cd4
Fcer1g
UMAP_1
UMAP_2
CD8
CD4
NK
Unidentified
ISAG
Dysfunctional
Terminal Effector
TRM
Inflammatory
Naive
HALLMARK_INTERFERON_ALPHA_RESPONSE
HALLMARK_INTERFERON_GAMMA_RESPONSE
HALLMARK_ALLOGRAFT_REJECTION
p<0.0001
p<0.001
p<0.01
Running Enrichment Score
Position in the Ranked List of Genes
IL2 STAT5 SIGNALING
DNA REPAIR
OXIDATIVE PHOSPHORYLATION
COMPLEMENT
ANGIOGENESIS
ALLOGRAFT REJECTION
INTERFERON GAMMA RESPONSE
INTERFERON ALPHA RESPONSE
Normalized Enrichment Score

Figure S5

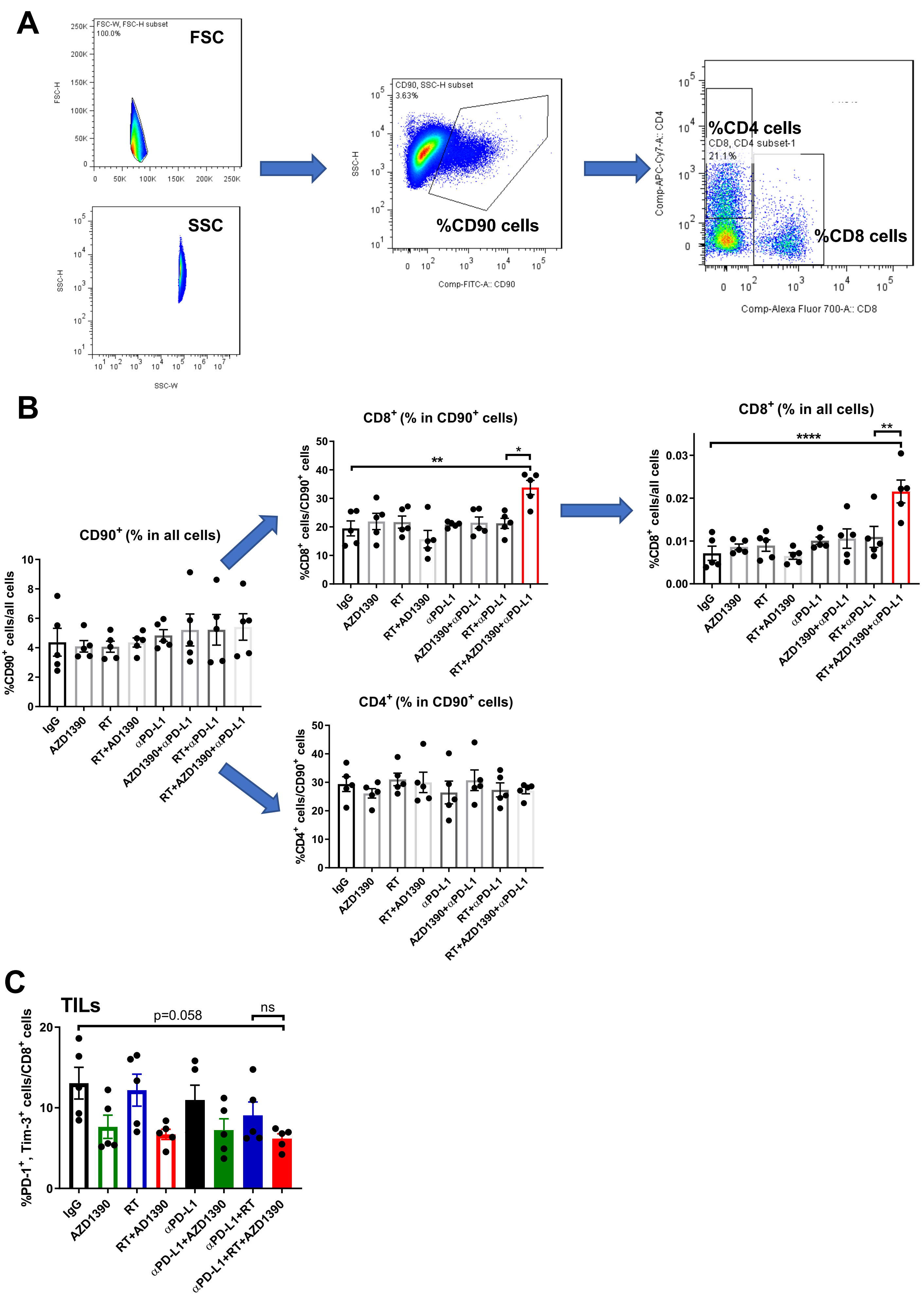

A
FSC
FSC-W, FSC-H subset
100.0%
SSC
CD90, SSC-H subset
3.63%
%CD90 cells
Comp-FITC-A:: CD90
%CD4 cells
CD8, CD4 subset-1
21.1%
%CD8 cells
Comp-APC-Cy7-A:: CD4
Comp-Alexa Fluor 700-A:: CD8
B
CD90+ (% in all cells)
%CD90+ cells/all cells
CD8+ (% in CD90+ cells)
%CD8+ cells/CD90+ cells
CD8+ (% in all cells)
%CD8+ cells/all cells
CD4+ (% in CD90+ cells)
%CD4+ cells/CD90+ cells
IgG
AZD1390
RT
RT+AD1390
αPD-L1
AZD1390+αPD-L1
RT+αPD-L1
RT+AZD1390+αPD-L1
C
TILs
p=0.058
ns
%PD-1+, Tim-3+ cells/CD8+ cells
αPD-L1+AZD1390
αPD-L1+RT
αPD-L1+RT+AZD1390

Figure S6

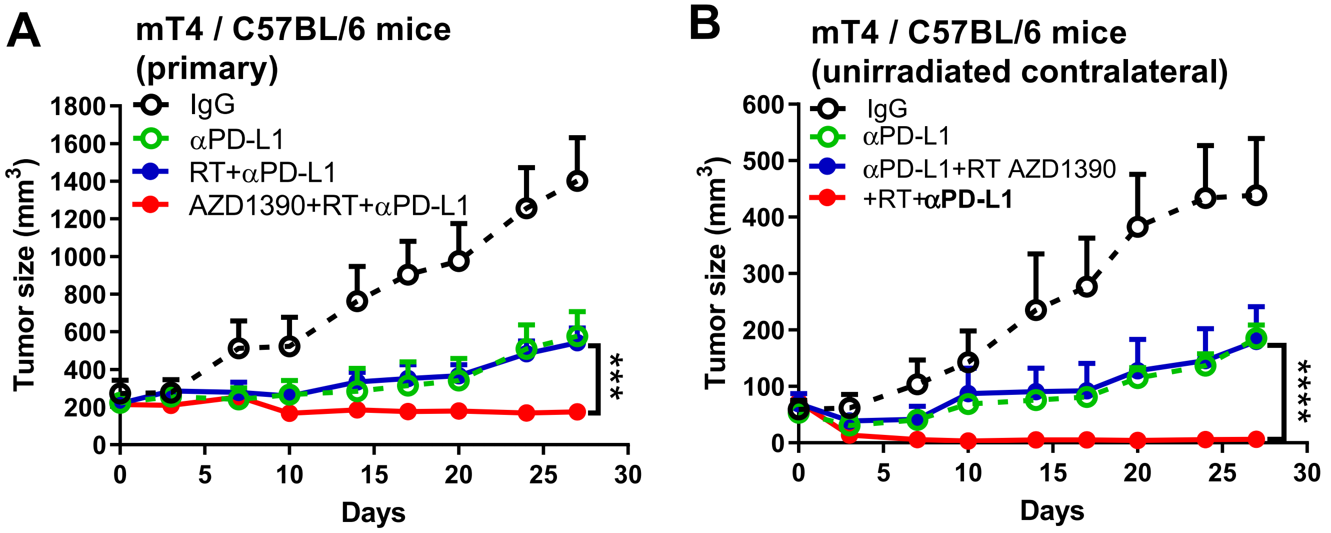

A
mT4 / C57BL/6 mice
(primary)
IgG
αPD-L1
RT+αPD-L1
AZD1390+RT+αPD-L1
Tumor size (mm3)
Days
***
B
mT4 / C57BL/6 mice
(unirradiated contralateral)
IgG
αPD-L1
αPD-L1+RT AZD1390
+RT+αPD-L1
Tumor size (mm3)
Days
****

Supplementary Figure Legends

Figure S1. ATM inhibitor enhances radiation-induced T1IFN expression and signaling in a TBK1-dependent and cGAS/STING-independent manner. (**A, B**) qPCR analysis of *Cxcl9* (A) and *Cxcl10* (B) mRNA levels in murine KPC2 cells at 3 days after treatment with radiation (8 Gy) and/or treatment AZD0156 (30 nM) or AZD1390 (30 nM). (**C**) Flow cytometry analysis of cell surface PD-L1 expression in KPC2 cells with the indicated treatments. Data are the MFI for PD-L1 minus the MFI for isotype control and shown as relative fold change. (**D**) Immunoblot analysis of TBK1 phosphorylation levels in Ctrl, cGAS, and STING-deleted Panc1 cells following herring testis (HT)-DNA transfection (1 µg/ml, 4 hr). (**E**) GFP MFI in indicated (sgCtrl, sgSTING, or sgcGAS) Panc1-*IFNβ1* promotor-GFP reporter cells at 3 days following radiation (8 Gy) and/or AZD0156 (30 nM) or AZD1390 (30 nM). (**F**) qPCR for *IFNβ1* in indicated (sgCtrl, sgSTING, or sgcGAS) Panc1 cells 3 days after treatment with radiation (8 Gy) and/or AZD0156 (30 nM) or AZD1390 (30 nM). (**G**) Cell surface PD-L1 in Panc1 cells (sgCtrl, sgSTING, or sgcGAS) at 3 days following treatment with and/or radiation (8 Gy) and AZD0156 (30 nM) or AZD1390 (30 nM) 1hr before radiation. Data are the MFI for PD-L1 minus the MFI for isotype control and shown as relative fold change. (**H**) Ctrl and TBK1-KO *IFNβ1* promoter-GFP reporter Panc1 cells treated with radiation (8 Gy) and/or AZD0156 (30 nM) or AZD1390 (30 nM) and tested MFI of GFP expression at Day 3. (**I**) qPCR analysis of *IFNβ1* mRNA levels in Ctrl and TBK1-KO Panc1 cells treated with radiation (8 Gy) and/or AZD0156 (30 nM) or AZD1390 (30 nM) at Day 3. (**J**) Flow cytometry analysis of cell surface PD-L1 in Panc1 cells (sgCtrl, sgTBK1) at 3 days following treatment with radiation (8 Gy) and/or AZD0156 (30 nM) or AZD1390 (30 nM). (**K**) Ctrl, cGAS, and STING knockout Panc1 cells with stable *IFNβ1* promoter-GFP reporter treated with radiation (8 Gy) and/or AZD1390 (30 nM), AZD6738 (200 nM), or M3814 (100 nM) and tested MFI of GFP expression at Day 3. In plots A-C and E-K, data are the mean ± SEM (n=3 independent experiments with each performed in technical triplicate). Statistical analyses were carried out by

one-way ANOVA with a multiple comparison post-test; *P < 0.05, **P < 0.01, ***P < 0.001, ****P< 0.0001.

Figure S2. Inhibition of RNA POLIII or depletion of RIG-I and MAVS blocks ATM inhibitor and radiation-induced interferon stimulated gene expression. (**A**, **B**) *Cxcl9* and *Cxcl10* mRNA levels were measured by qPCR in KPC2 cells treated with radiation (8 Gy) and/or AZD1390 (30 nM) in the absence or presence of the POLIII inhibitor ML-60218. (**C**, **D**) Ctrl and ML-60218 treated Panc1 (C) and KPC2 (D) cells following treatment with radiation (8 Gy) and/or AZD1390 (30 nM) were analyzed for cell surface PD-L1 expression by flow cytometry. (**E**) His-HA-ubiquitin transfected Panc1 cells were treated with radiation (8 Gy) and/or AZD0156 (30 nM) or AZD1390 (30 nM) for 6 hours. Ubiquitination assay was performed to determine endogenous RIG-I ubiquitination using Ni-NTA beads and Western blot analysis. (**F**) Representative cell cycle analysis of shCtrl, shRIG-I, and shMAVS Panc1 cells at 24 hours following radiation (8 Gy) and/or AZD1390 (30 nM) with staining for DNA content (PI, x-axis) and phosphorylated-Histone H3 (Ser10; y-axis). (**G**) Ctrl, cGAS, and STING knockdown Panc1 cells with stable *IFNβ1* promoter-GFP reporter treated with radiation (8 Gy) and/or AZD1390 (30 nM), AZD6738 (200 nM), or M3814 (100 nM) and tested MFI of GFP expression at day 3. Data are the mean ± SEM with 2-3 independent experiments with each performed in technical triplicate. Statistical analyses were carried out by one-way ANOVA with a multiple comparison post-test; *P < 0.05, **P < 0.01, ***P < 0.001, ****P< 0.0001.

Figure S3. Combined therapy with ATM inhibitor, radiotherapy, and anti-PD-L1 inhibits pancreatic tumor growth and induces durable anti-tumor immune responses. (**A**) Individual tumor volumes for data shown in Figure 3B. Number of mice per (treatment arm) = 8 (ctrl), 10 (AZD1390), 14 (RT), 16 (AZD1390+RT), 12 (αPD-L1), 16 (αPD-L1+AZD1390), 16 (αPD-L1+RT), and 20 (αPD-L1+AZD1390+RT). (**B**) Body weight of the mice shown in Figure 3B. (**C, D**) FVB mice with KPC2 tumors were treated as illustrated (Figure 3A). Data are the mean tumor volume

± SEM (B) or tumor volume doubling time (C). Data are from n = 10 (ctrl), 10 (AZD1390), 12 (αPD-L1), 16 (AZD1390+αPD-L1), 14 (RT), 16 (AZD1390+RT), 16 (αPD-L1+RT), and 20 (AZD1390+RT αPD-L1) tumors per treatment group. (**E**) Individual tumor volumes for data shown in Figure S3C. (**F**) Mice with complete responses to AZD1390, RT, and αPD-L1 were rechallenged with KPC2 (10^6) cells 7 days after complete response. Naïve FVB mice were similarly rechallenged. Data are the mean tumor volume from naïve (n = 10) or previously treated FVB (n=4). In Panel B, C, and F, data are the mean ± SEM. Statistical analyses were carried out by one-way ANOVA with a multiple comparison post-test; **P < 0.01, ****P< 0.0001.

Figure S4. ATM inhibitor, radiotherapy, and anti-PD-L1 treatment alters the tumor immune microenvironment. (**A**) Cell specific markers used to identify clusters of cells from harvested subcutaneous mT4 tumors. (**B**) Interferon stimulated gene expression in AZD1390, RT+αPD-L1, or AZD1390+RT+αPD-L1 treatments compared to control. (**C**) Identification of *Cd8a* expressing cells from the T cell cluster that were used to subset and re-cluster. (**D**) $CD8^+$ T cell specific markers used to identify clusters. (**E**) GSEA plot of interferon alpha and gamma responses, and allograft rejection based on gene expression in T cells from combined therapy with radiation and anti-PD-L1. (**F**) GSEA normalized Hallmark pathways associated with AZD1390/RT/αPD-L1 treatment in $CD8^+$ T cells. Statistical significance in E was determined using an unpaired t-test. P value indicated.

Figure S5. AZD1390, radiation, and αPD-L1 increase tumor-infiltrated $CD8^+$ T, but not $CD4^+$ T cell numbers. (**A**) Gating strategy to determine $CD90^+$, $CD4^+$, and $CD8^+$ cells in the mT4 tumors treated with AZD1390, radiation, and/or αPD-L1 (as illustrated in Fig 3A) at day 10. (**B**) Quantitation of the percentage of tumor infiltrating lymphocyte $CD4^+$ (in the $CD90^+$ cell population) and $CD8^+$ cell numbers (in the $CD90^+$ and total cell population respectively) stained at day 10. Data are the mean ± SEM. (**C**) Flow cytometry for the percentage of $PD1^+$, $Tim3^+$ positive tumor $CD8^+$ cells in mT4 tumors treated with AZD1390, radiation, and/or αPD-L1 (as illustrated in Fig

3A) and harvested at day 10. Statistical analyses were carried out by one-way ANOVA with a multiple comparison post-test; *P < 0.05, **P < 0.01, ****P< 0.0001.

Figure S6. ATM inhibition, radiotherapy and αPD-L1 exhibit systemic tumor control. (A, B) Tumor growth curves of primary tumors (A) and unirradiated contralateral tumors (B) in mT4 tumor-bearing mice after the indicated treatments. Number of mice per (treatment arm) = 5 (ctrl), 7 (αPD-L1), 8 (αPD-L1+RT), and 10 (αPD-L1+AZD1390+RT). Data are the mean tumor volume ± SEM. Statistical analyses were carried out by one-way ANOVA with a multiple comparison post-test; ***P < 0.001, ****P< 0.0001.
