# Supplementary material for: Potentiating the radiation-induced type I interferon antitumoral immune response by ATM inhibition in pancreatic cancer
Source: JCI Insight. 2024 Feb 20;9(6):e168824. doi: 10.1172/jci.insight.168824 (PMC11063931; doi:10.1172/jci.insight.168824)
Supplement: Unedited blot and gel images [file jciinsight-9-168824-s107.pptx]

## Slide 1
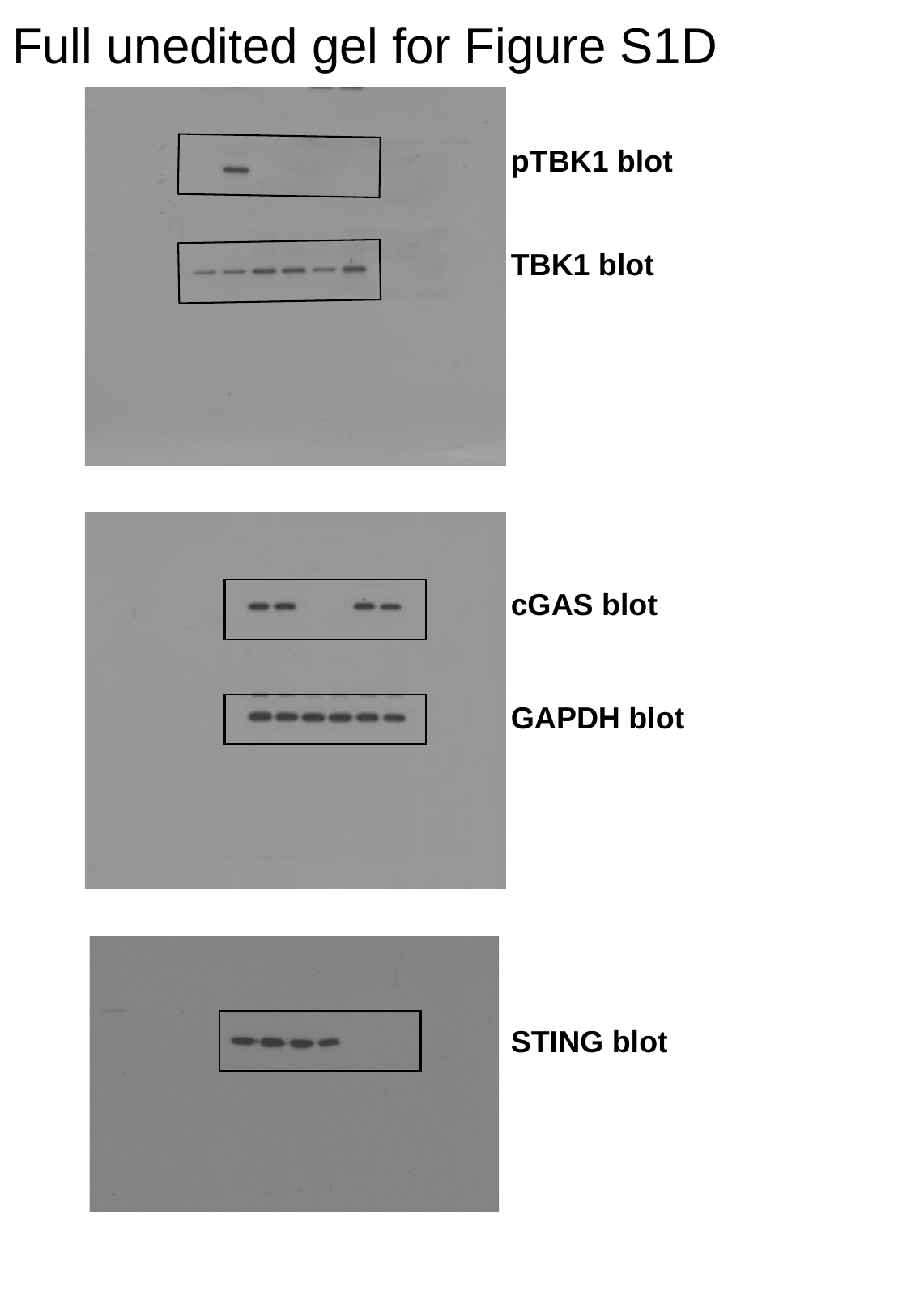

# Full unedited gel for Figure S1D
pTBK1 blot
TBK1 blot
cGAS blot
GAPDH blot
STING blot

## Slide 2
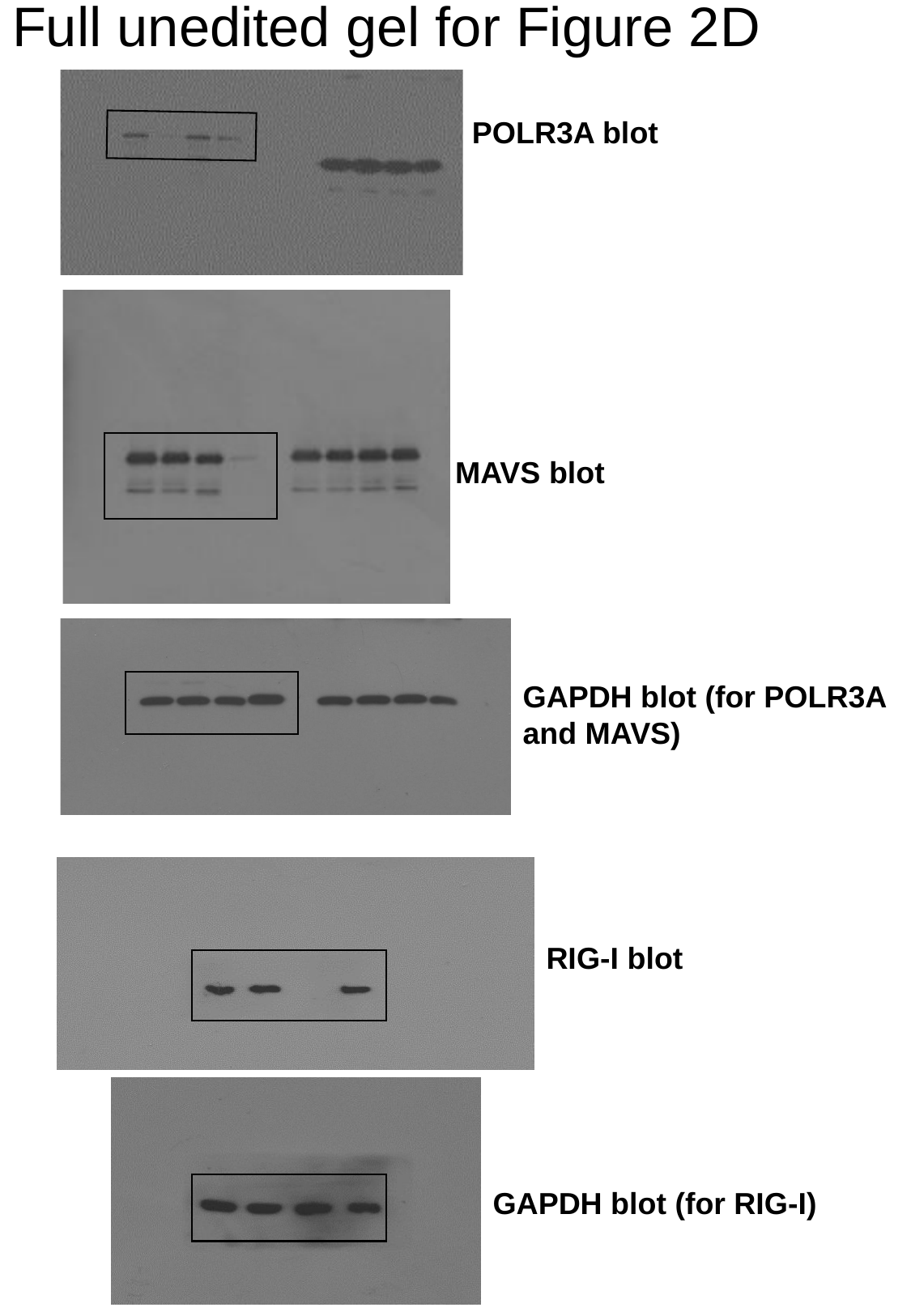

# Full unedited gel for Figure 2D
POLR3A blot
MAVS blot
GAPDH blot (for POLR3A and MAVS)
RIG-I blot
GAPDH blot (for RIG-I)

## Slide 3
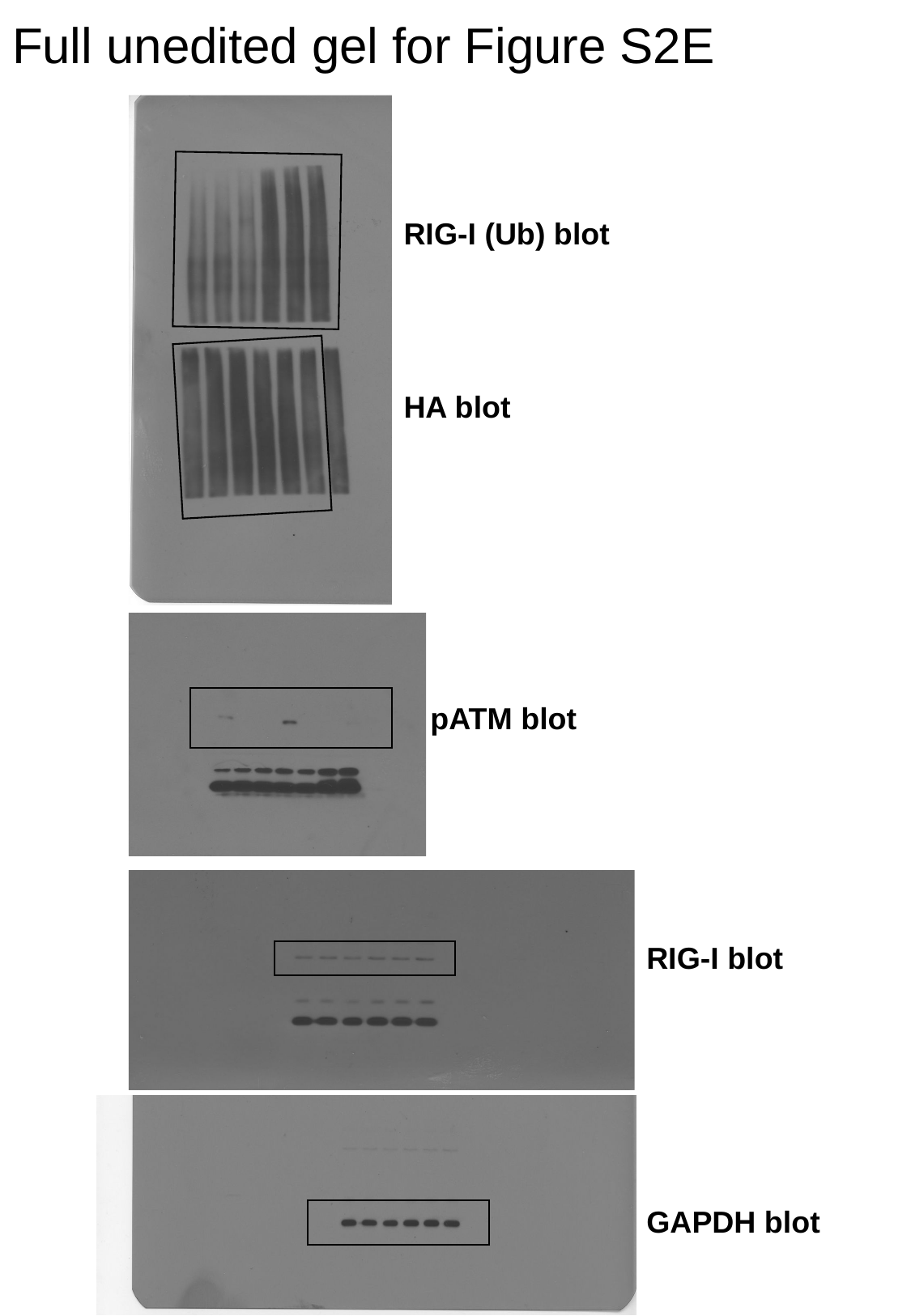

# Full unedited gel for Figure S2E
RIG-I (Ub) blot
HA blot
pATM blot
RIG-I blot
GAPDH blot
